# Identification of the S100 fused-type protein hornerin as a regulator of tumor vascularity

**DOI:** 10.1038/s41467-017-00488-6

**Published:** 2017-09-15

**Authors:** Michael F. Gutknecht, Marc E. Seaman, Bo Ning, Daniel Auger Cornejo, Emily Mugler, Patrick F. Antkowiak, Christopher A. Moskaluk, Song Hu, Frederick H. Epstein, Kimberly A. Kelly

**Affiliations:** 1 Cardiovascular Research Center, The University of Virginia, Box 801394, Charlottesville, Virginia 22908 USA; 2 Department of Biomedical Engineering, The University of Virginia, Box 800759 Health System, Charlottesville, Virginia 22908 USA; 3 Department of Pathology, The University of Virginia, Box 800904, Charlottesville, Virginia 22908 USA; 4 Department of Biochemistry and Molecular Genetics, The University of Virginia, Box 800733, Charlottesville, 22908 Virginia USA

## Abstract

Sustained angiogenesis is essential for the development of solid tumors and metastatic disease. Disruption of signaling pathways that govern tumor vascularity provide a potential avenue to thwart cancer progression. Through phage display-based functional proteomics, immunohistochemical analysis of human pancreatic ductal carcinoma (PDAC) specimens, and in vitro validation, we reveal that hornerin, an S100 fused-type protein, is highly expressed on pancreatic tumor endothelium in a vascular endothelial growth factor (VEGF)-independent manner. Murine-specific hornerin knockdown in PDAC xenografts results in tumor vessels with decreased radii and tortuosity. Hornerin knockdown tumors have significantly reduced leakiness, increased oxygenation, and greater apoptosis. Additionally, these tumors show a significant reduction in growth, a response that is further heightened when therapeutic inhibition of VEGF receptor 2 (VEGFR2) is utilized in combination with hornerin knockdown. These results indicate that hornerin is highly expressed in pancreatic tumor endothelium and alters tumor vessel parameters through a VEGF-independent mechanism.

## Abstract (web summary)

Angiogenesis is essential for solid tumor progression. Here, the authors interrogate the proteome of pancreatic cancer endothelium via phage display and identify hornerin as a critical protein whose expression is essential to maintain the pancreatic cancer vasculature through a VEGF-independent mechanism.

## Introduction

The generation and maintenance of a tumor vascular network is essential for tumor growth and provides a conduit for tumor cell metastasis. In their seminal paper, Hanahan and Weinberg^1^ listed sustained angiogenesis as one of the original hallmarks of cancer. Research in the intervening years has bolstered the significance of this process and necessitated its inclusion in their follow-up paper a decade later^2^. As vessels provide the fuel for a growing tumor, anti-angiogenic therapies designed to impede the rapid, dysregulated propagation of vessels or enhance endothelial cell death have been proposed and at times implemented in clinic. These are commonly combined with anti-cancer cell therapies, resulting in an overall strategy that targets critical signaling pathways in different cell types that comprise the tumor microenvironment^3, 4^.

The discovery of vascular endothelial growth factor (VEGF), originally called vascular permeability factor, in 1983^5^ dramatically increased the knowledge of angiogenesis in many fields, including tumor angiogenesis. Since it is extensively studied and highly overexpressed in cancer as well as other pathologies, the VEGF pathway was a natural initial target when developing anti-angiogenesis therapies. Anti-VEGF therapies include small molecule inhibitors, VEGF receptor blocking antibodies, and VEGF traps, which mimic the receptor-binding site and sequester VEGF from its receptor. In a review of anti-angiogenic therapies in pancreatic cancer, Whipple et al.^6^ listed 12 anti-angiogenic therapies that are in use for this type of cancer alone. When browsing the list, it is striking that 10 of those 12 therapies target the VEGF pathway. Anti-angiogenic (mostly anti-VEGF) therapies have achieved success pre-clinically and clinically, however, as is common with monotherapies, tumor resistance and recurrence is a major problem.

One hypothesis for the lack of success could be the presence of compensatory or redundant pathways. Compensatory pathways are well established in tumor therapy research; consequently clinical trials devoted to synergistic combinations of drugs have proliferated^7–9^. Combinatorial approaches involving non-redundant signaling pathways show great promise, as they have the ability to overcome acquired resistance to chemotherapy^10^. A synergistic/additive approach, one involving both the VEGF pathway and another important pathway, could provide the positive results that anti-angiogenic therapies should theoretically produce.

Therefore, to identify non-VEGF-mediated tumor angiogenic factors, we used a phage display functional proteomics approach that combined an in vivo phage screen in tumor-bearing animals with an in vitro screen to exclude clones that bound to VEGF-treated cells. Binding partner identification from this screen revealed the protein hornerin, which to this point had been studied predominantly in the skin epithelium^11–14^. Hornerin was further validated in human umbilical vein endothelial cells (HUVECs) as a non-VEGF regulated protein. While confirming hornerin expression in tumor-associated endothelial cells in resected human pancreatic ductal adenocarcinoma (PDAC) samples, it was discovered that hornerin is also expressed in PDAC. Further exploration identified several other tumor types that express hornerin, including renal cell carcinoma (RCC) and prostate adenocarcinoma. A functional role of hornerin was affirmed, as in vivo-specific knockdown of hornerin in tumor-associated endothelial cells resulted in decreased tumor burden along with alterations in vessel parameters as measured by vessel radius, vessel volume fraction, and fractal dimension. In addition, magnetic resonance imaging (MRI) of tumors with vessels with decreased hornerin expression revealed a decrease in vascular leakiness. Finally, hornerin knockdown combined with VEGF inhibition produced additive tumor volume and angiogenesis abatement, providing further evidence that compensatory pathways exist in tumor-associated endothelial cells. The discovery of elevated hornerin expression in tumor vasculature, the functional consequences of hornerin targeted knockdown on tumor vascularity and growth, and the additive effect of hornerin depletion with VEGFR signaling inhibition directs the potential creation of a novel anti-angiogenesis strategy that targets multiple signaling pathways in tumor endothelial cells.

## Results

### VEGF-independent vascular binding peptides

**Fig. 1:**
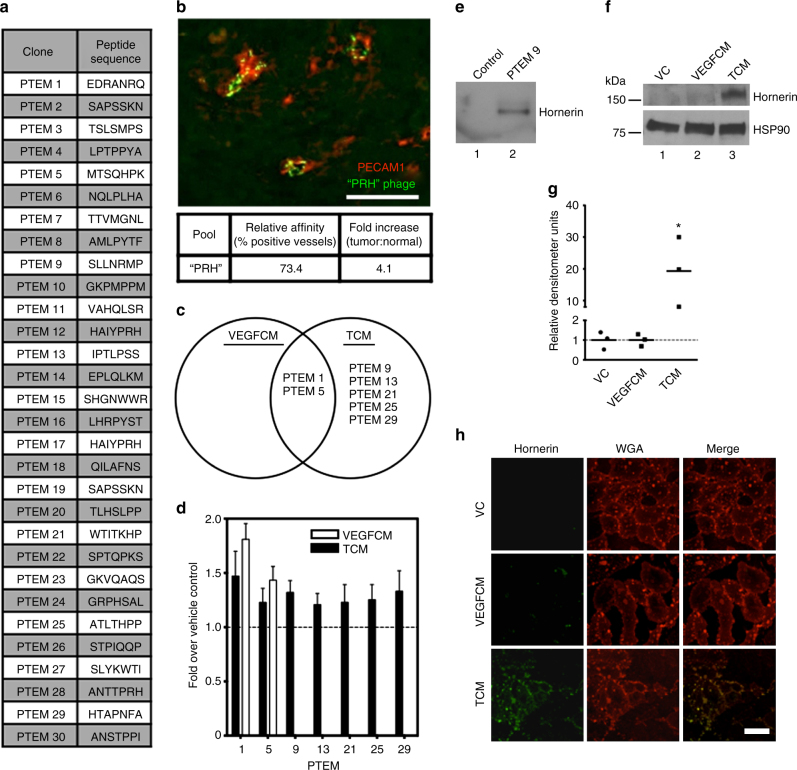
Identification of tumor endothelial cell-specific phage clones and hornerin as a non-VEGF but TCM-induced protein. **a** The amino-acid sequences of 30 selected phage clones that displayed selective binding to pancreatic tumor endothelium. **b** Orthotopic pancreatic tumor section showing co-localization of FITC-labeled “PRH” motif phage clones (*green*) with PCAM1-positive vessels (*red*). Relative affinity and fold increase over normal (adjacent, unaffected pancreas vessels) of the “PRH” pool is presented below the image. **c** Venn diagram generated after phage-based ELISA and two-way ANOVA analysis that illustrates specificity of phage clone binding to VEGFCM- and/or TCM-treated HUVECs. **d** Quantification of the specificity (fold over vehicle control (VC)) for each clone in the Venn diagram. *N* = 6 for each clone. **e** Immunoblot detection of hornerin in lysates generated from M13KE (control; lane 1) and PTEM 9 (lane 2) phage pulldowns. **f** Representative immunoblot of lysate preparations from VC (lane 1), VEGFCM (lane 2), and TCM (lane 3)-treated HUVECs display the levels of hornerin protein. HSP90 is shown as a loading control. Molecular weight is indicated in kilodaltons (kDa). **g** Relative densitometry measured as hornerin fold expression over HSP90. *N* = 3 for each condition. **h** Immunofluorescence detection of hornerin (column 1) on non-permeabilized HUVECs following treatment with VC (*top row*), VEGFCM (*middle row*), or TCM (*bottom row*). Wheat germ agglutinin (column 2) was included in the stain to highlight the plasma membrane. The merged image (column 3) displays hornerin and WGA co-localization. *Scale bar* = 50 μm. Graphs represent the mean ± SEM. *P ≤ .05 by unpaired two-tailed *t*-test, treatment compared to vehicle control

To elucidate peptides that bind specifically to tumor endothelium, an in vivo phage display screen was performed in mice bearing orthotopically implanted human PDAC cells. After three rounds of selection, 30 phage clones that we termed pancreatic tumor endothelial markers (PTEM) were sequenced (Fig. 1a). Before continuing with the in vitro screen, we wanted to assess the specificity of a few of the selected clones for blood vessels in vivo through injection of fluorescently labeled phage^15^ into animals bearing orthotopic tumors. Specificity of the phage for tumor vessels was assessed by immunofluorescence (Fig. 1b). We used three clones with similar homology, PRH, in our analysis. The “PRH” pool (PTEM 12/17, 16) bound 73.4% (4.1-fold over normal pancreas vessels) of platelet endothelial cell adhesion molecule1-positive tumor vessels (Fig. 1b). The immunofluorescent images indicate that the fluorescein isothiocyanate (FITC)-phage remained bound to the luminal surface of the blood vessels even after vigorous perfusion.

As VEGF is highly abundant in the pancreatic tumor milieu, we decided to further segregate clones based on their ability to bind to VEGF-treated HUVECs vs. tumor conditioned media (TCM)-treated HUVECs. HUVECs were utilized as we were interested in the ability of the peptides to bind to both human and mouse antigens. Enzyme-linked immunoabsorbent assay (ELISA) results were analyzed by two-way analysis of variance (ANOVA) to find clones that consistently bound to HUVECs treated with TCM, but not vehicle control (VC) or VEGF-supplemented control media (VEGFCM) (Supplementary Fig. 1a, b). After analysis, the clones were grouped and organized in a Venn diagram (Fig. 1c). Although the PRH family demonstrated specificity and selectivity for tumor blood vessels in vivo, it failed to meet these criteria in subsequent in vitro screens, suggesting that it does not cross-react with a human target or the target is not present on human cells. Of the clones that bound selectively to TCM-treated HUVECs, PTEM 9 demonstrated the highest average specificity (Fig. 1d). These data were further validated in a subsequent ELISA using both M13KE phage (no displayed peptide; control) and PTEM 9 phage (Supplementary Fig. 1c).

### Hornerin identified as the binding partner of PTEM 9

We next employed phage display-based functional proteomics to determine the binding partner of PTEM 9^16^. Tandem mass spectroscopy sequencing of lysates resulting from the functional proteomics process revealed hornerin as the candidate protein; six unique tryptic digest fragments were identified resulting in a percent coverage of 3.5%, coverage similar to what has been previously published utilizing this method (Supplementary Fig. 2a, b; Supplementary Data 1)^16, 17^. To confirm that hornerin was the identified protein, we performed anti-hornerin immunoblotting on the PTEM 9 phage and control M13KE phage lysates. A unique band in the PTEM 9 lane that was not present in the control sample corresponded to the predicted molecular weight of hornerin (Fig. 1e).

### Non-VEGF soluble factors increase hornerin expression

Based on the strategy utilized to segregate the peptides presented in Fig. 1c and Supplementary Fig. 1, we hypothesized that expression of hornerin in HUVECs would increase upon treatment with TCM but not in response to VEGF. To test this, cell lysates from HUVECs treated with TCM, VEGFCM, and VC media were analyzed by immunoblot for the expression of hornerin. In TCM-treated HUVECs, hornerin expression was 19.3-fold higher when compared with VC, while treatment of HUVECs with VEGFCM resulted in no significant change of hornerin expression over VC (Fig. 1f, g). Immunofluorescent detection of hornerin in non-permeabilized HUVECs suggests hornerin co-localization with the plasma membrane following treatment with TCM (Fig. 1h). These data strongly indicate that hornerin is upregulated in response to factor(s) in the TCM and that hornerin accumulates at the plasma membrane.

### Hornerin is expressed in human PDAC vessels

**Fig. 2:**
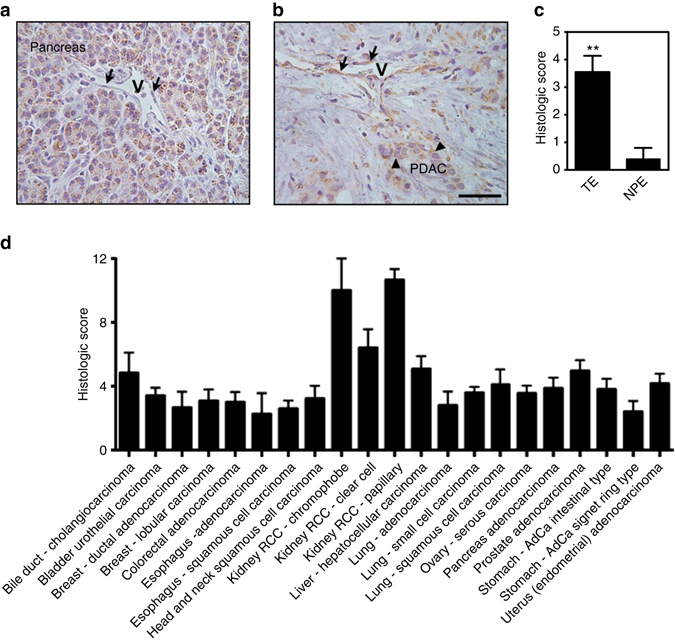
Comparative hornerin expression in normal pancreas and resected human PDAC tumors. Images of **a** normal unaffected pancreas and vessels (designated as “V”) and **b** PDAC (*arrowheads*) and associated vessels (“V”) following anti-hornerin immunohistochemistry. Endothelial cells are marked with *black arrows* in **a**, **b**. **c** Overall histologic score of tumor endothelium (TE) (*N* = 10) and normal pancreas endothelium (NPE) (*N* = 5). **d** Hornerin was detected by immunohistochemistry in tumor microarray specimens and scored by a certified pathologist. *N* = 2–12/tumor type. *Scale bar* = 100 μm. Graphs represent the mean ± SEM. **P < 0.005 by unpaired two-tailed *t*-test

We next sought to determine if hornerin is expressed in human tumor specimens. Hornerin expression in specimens from 10 PDAC patients (Supplementary Table 1) was revealed by immunohistochemistry and scored by a certified pathologist at the University of Virginia. Normal pancreas endothelial cells (NPE) (*arrows*) were negative for hornerin expression (Fig. 2a), however tumor endothelial cells (TE) (*arrows*) near PDAC (*arrowheads*) stained positively (Fig. 2b). Importantly, 5 of the 10 resected PDAC samples had areas of non-affected pancreas where NPE staining could be quantified. Analysis of the samples in each scoring component (intensity and percent positively stained cells; Supplementary Fig. 3a, b) revealed a large disparity between NPE and TE, with TE having a 7.1-fold higher score than NPE (Fig. 2c).

We observed that PDAC cells and normal pancreas cells also stain positive for hornerin, indicating that hornerin may not be a good immunohistochemical biomarker for PDAC. However, the increased vessel expression suggested a tumor-specific phenotype and prompted the studies presented in this report. To expand our analysis of hornerin expression in human tumor specimens, a pathologist scored several types of solid tumors in addition to PDAC. Notably, RCC (chromophobe, clear cell, and papillary) displayed the highest relative expression of hornerin in the panel we evaluated (Fig. 2d).

### Hornerin knockdown leads to decreased tumor burden

**Fig. 3:**
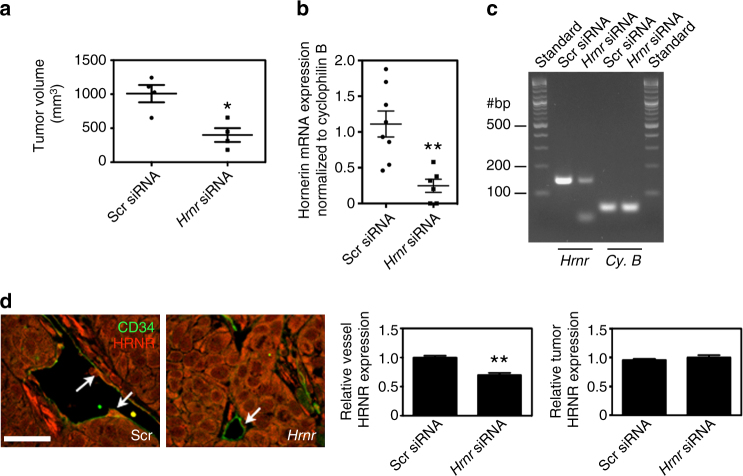
siRNA-mediated hornerin knockdown results in decreased tumor burden. **a** L3.6pl PDAC cells were injected subcutaneously into the mouse flank and tumor volume was determined upon termination of the experiment (day 13 post implantation). Tumors were injected with either mouse hornerin (*Hrnr* siRNA) or scrambled control (Scr siRNA) siRNA every other day starting on day 7. Graph displays the mean calculated tumor volume derived from caliper measurements ((tumor length × tumor width^2^)/2). *N* = 4 tumors/group. **b** RNA was isolated from FACS-sorted CD31^+^CD45^−^ tumor endothelial cells and subjected to qPCR. The graph displays the normalized hornerin mRNA transcript levels (reference gene cyclophilin B) from Scr siRNA- and *Hrnr* siRNA-treated mice. The data points represent sorted endothelial cell sample preparations from two independent experiments run in quadruplicate. *N* = 8 (Scr siRNA), 6 (*Hrnr* siRNA). **c** End-point qPCR samples from the above reaction were resolved on a 4% agarose gel and imaged for SYBR green intensity. Gel represents reaction samples following hornerin (*Hrnr*) and cyclophilin B (*Cy*. *B*) primer set amplification, respectively. The predicted size for the hornerin amplicon is 149 base pairs. #bp = number of base pairs in the noted standard bands. **d** Representative images of CD34-positive vessels (*green*) and hornerin (*red*) in Scr (*left*) or *Hrnr* (*right*) siRNA-injected tumors. Endothelial cells of note are highlighted with *arrows*. Graphs display the mean expression of hornerin in the CD34^+^ endothelial cells (*left graph*) and tumor (*right graph*). *Scale bar* = 50 μm. Graphs represent mean ± SEM. *P < 0.05, **P < 0.005 by unpaired two-tailed *t*-test

As hornerin is present in tumor vessels but not normal vessels, we hypothesized that hornerin may play an important role in tumor vessel function and consequently regulate tumor progression. To address this, we decreased endothelial hornerin expression using intratumoral injections of mouse-specific hornerin siRNA (*Hrnr* siRNA) in a subcutaneous xenograft PDAC model (L3.6pl; PDAC of human origin). We chose this model system carefully such that epithelial tumor tissue would be unaffected by *Hrnr* siRNA knockdown. To confirm that the *Hrnr* siRNA does not target human *HRNR*, we conducted in vitro siRNA knockdown experiments in mouse epidermal keratinocytes (COCA) and human keratinocytes. These cell lines were selected based on previous studies showing elevated hornerin expression in the epidermis^12–14^. Our results indicated a 60% reduction in hornerin expression in COCA cells treated with *Hrnr* siRNA relative to control scrambled siRNA (Scr siRNA)-treated cells (Supplementary Fig. 4a). Loss of hornerin was not observed to a significant degree (~10%) in the human keratinocytes under similar conditions, thus providing support that the *Hrnr* siRNA utilized in our studies is mouse-specific (Supplementary Fig. 4b). Remarkably, intratumoral injections of *Hrnr* siRNA resulted in tumors that were 2.5-fold smaller compared to tumors that were injected with Scr siRNA (Fig. 3a). To confirm hornerin knockdown in the tumor endothelium, we performed quantitative PCR (qPCR) on fluorescence-activated cell sorting (FACS) sorted tumor CD31^+^CD45^−^ cells and observed a marked 78% reduction in hornerin transcript in the *Hrnr* siRNA-treated cohort (Fig. 3b). Analysis of qPCR end-point samples revealed an intense band at the predicted amplicon size (149 base pairs), indicating that our primers were specific for the intended target (Fig. 3c). To provide further support for endothelial cell-hornerin knockdown and targeted siRNA specificity for murine and not human hornerin, we analyzed tumor sections for hornerin protein expression by immunofluorescent microscopy. Tumor vessels treated with Scr siRNA maintained high expression of hornerin, while hornerin expression in *Hrnr* siRNA-treated vessels was greatly reduced (Fig. 3d). Quantitation of sections revealed no difference between the expression of hornerin in epithelial cells from tumors treated with either Scr siRNA or *Hrnr* siRNA. In contrast, there was a 30% decrease in hornerin expression in the endothelial cells between the two treatments (Fig. 3d). Antibody cross-reactivity to mouse hornerin was confirmed through indirect immunofluorescent microscopy in COCA cells and human keratinocytes (Supplementary Fig. 5).

### Hornerin knockdown alters key tumor vessel parameters

**Fig. 4:**
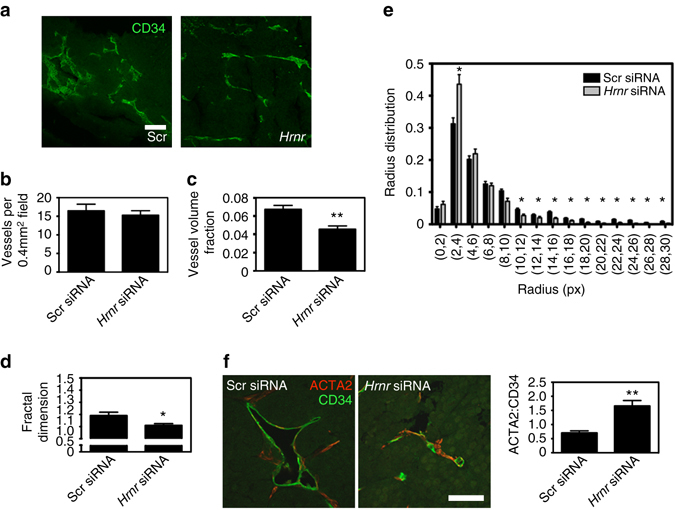
siRNA-mediated hornerin knockdown results in altered tumor vessel parameters. **a** Representative whole mount Z-stacked images of CD34^+^ tumor vessels (*green*) from Scr siRNA (*left*) and *Hrnr* siRNA (*right*) 50 µm tumor sections. **b** Graph represents the number of CD34-positive vessels per 0.4 mm^2^ field. Sections = 5 μm, *N* = 16/group (four tumors in each treatment group, four images per tumor). Vessel parameters including **c** vessel volume fraction, **d** fractal dimension, and **e** radius distribution in pixel units were determined from RAVE analysis of 14 *Hrnr* siRNA and Scr siRNAwhole-mount Z-stacks (four tumors in each treatment group, 3–4 images/tumor). **f** Representative images of actin, alpha-2 (ACTA2) (*red*) coverage on CD34^+^ tumor vessels (*green*) in tumor sections from Scr siRNA (*left*) and *Hrnr* siRNA (*right*) -treated mice. Graph represents the ratiometric quantification, as determined by dividing ACTA2-positive pixels by CD34-positive pixels, of 18 *Hrnr* siRNA sections (four tumors, 3–5 sections per tumor) and 20 Scr siRNA sections (four tumors, five sections per tumor). *Scale bar* **a** = 100 μm, **f** = 50 μm. Graphs represent mean ± SEM. *P < 0.05, **P < 0.005 by unpaired two-tailed *t*-test

We next used the matrix laboratory (MATLAB)-based vessel analysis software program rapid analysis of vessel elements (RAVE)^18^ to measure vessel volume fraction (VVF), fractal dimension (a measure of tortuosity), and vessel radius, parameters commonly evaluated in tumor vasculature^19^. Immunofluorescent analysis of tumor sections revealed that vessels in *Hrnr* siRNA-injected tumors had drastic differences in appearance, notably smaller radius and length and reduced tortuosity (Fig. 4a). Interestingly, the number of vessels per field (Fig. 4b) and the proportion of endothelial cells (CD31^+^CD45^−^), as determined by FACS (Supplementary Fig. 6a, b), were not different. *Hrnr* siRNA injection reduced VVF and fractal dimension as well (Fig. 4c, d). Additionally, there was an overall reduction in vessel radius from larger to smaller vessels (Fig. 4e). To confirm that our results were not due to off target effects of *Hrnr* siRNA, we treated a second set of tumor-bearing mice with Scr siRNA or a pool of three additional mouse *Hrnr* siRNA and analyzed similar vessel parameters by immunofluorescent microscopy. We again observed a trend toward smaller vessel radii and a reduction in vessel volume and fractal dimension in the *Hrnr* siRNA group relative to control (Supplementary Fig. 7)

A mature vessel is typically highly invested with alpha-smooth muscle actin (ACTA2)-positive pericytes^19, 20^. Conversely, tumor vessels lack pericyte investment and evidence suggests that pericyte–endothelial interactions may play a role in vessel morphology and functionality in tumors^19^. Upon injection of endothelial-targeted *Hrnr* siRNA, we observed a 2.3-fold increase in ACTA2 coverage of vessels when compared with Scr siRNA (Fig. 4f), a result similar to previous reports^21^. The cumulative data suggest that hornerin is not required to maintain the number of tumor vessels, however important vessel parameters such as VVF, tortuosity, pericyte recruitment, and radius are altered by the downregulation of hornerin expression.

### Altered tumor vessel function with *Hrnr* siRNA treatment

**Fig. 5:**
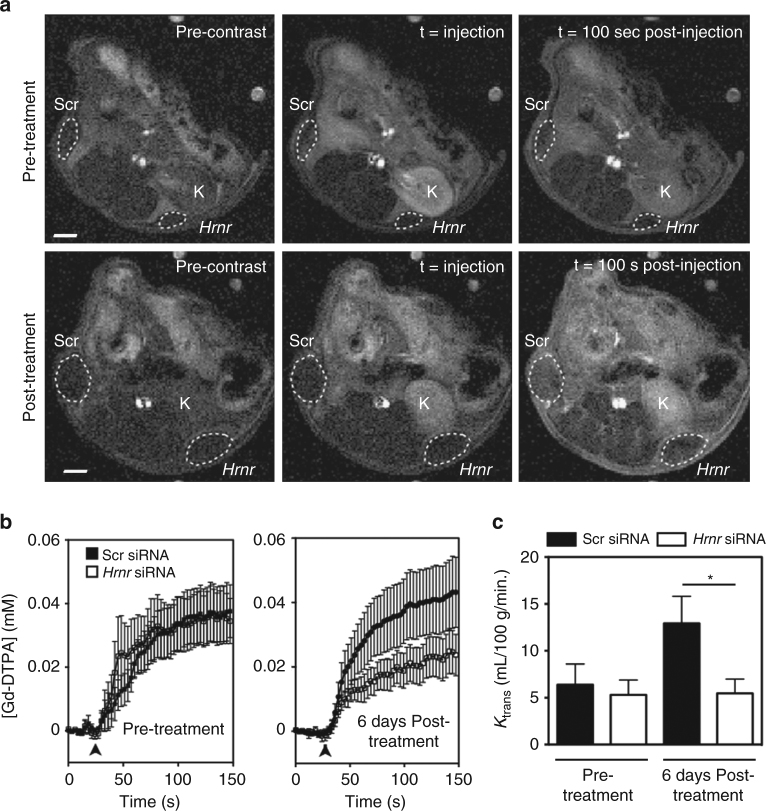
Dynamic contrast-enhanced magnetic resonance imaging reveals decreased vascularity and perfusion in *Hrnr* siRNA-treated mice compared to control mice. **a** Representative axial time course images of gadolinium-DTPA (Gd-DTPA) uptake taken pre-siRNA treatment (*top row*) and 6 days post-treatment (*bottom row*). The kidney (“K”) is labeled for reference. Scr siRNA and *Hrnr* siRNA tumors demarcated by a *white dashed line*. **b** The concentration of Gd-DTPA (mM) in the tumors was plotted over time. *N* = 7 tumors/treatment group. Injection of Gd-DTPA is signified by *arrowhead*. **c** *K*_trans_ was calculated for both pre-treatment and post-treatment tumors. *N* = 7 tumors/treatment group. *Scale bar* = 2 mm. Graphs represent mean ± SEM. *P < 0.05 by unpaired two-tailed *t*-test

Tumor vessels serve as an important supply line to a growing tumor. Consequently, disruption of this supply line should reduce tumor volume—the major hypothesis behind anti-angiogenesis therapies. Ex vivo vessel parameters such as vessel number, VVF, and vessel radius provide excellent insight into the structural and anatomic changes that occur as a result of therapeutic intervention; however, in vivo functional studies of tumor vascularity and perfusion are needed to determine if the delivery of oxygen and nutrients is actually reduced. Therefore, we treated tumors with Scr or *Hrnr* siRNA and measured functional changes through dynamic contrast-enhanced (DCE) MRI^22^. Mice were imaged before treatment to establish baseline levels of vascularity. No difference was observed in the pre-treatment images (Fig. 5a, *top row*) as determined by gadolinium-diethylenetriamine penta-acetic acid (contrast agent, Gd-DTPA) time intensity curves (Fig. 5b, *left*) or volume transfer coefficient (*K*_trans_) (Fig. 5c, *left*). After pre-treatment imaging, tumors were randomly assigned to Scr or *Hrnr* siRNA treatment groups. Although tumors did not receive treatment prior to pre-treatment scans, they are labeled as Scr or *Hrnr* to indicate their subsequent treatment designation in Fig. 5a (*lower row*), 5b (*right*), and 5c (*right*). Importantly, after just 6 days of treatment (siRNA injection every other day), large differences in Gd-DTPA accumulation and *K*_trans_ values were observed in Scr and *Hrnr* siRNA-injected tumors. After 100 s, Gd-DTPA signal intensity is elevated in the Scr siRNA-treated tumors compared to the *Hrnr* siRNA-treated tumors (Fig. 5a, third column; Supplementary Movie 1). The Gd-DTPA time intensity curves separate 15–20 s after injection and tumor concentration of Gd-DTPA remains two-fold reduced in *Hrnr* siRNA-injected tumors through the entire time course (Fig. 5b, *right*). Likewise, after 6 days of siRNA injections, a 2.4-fold reduction in *K*_trans_ is observed in hornerin knockdown tumors compared to control (Fig. 5c, *right*).

As a control, we used a VEGFR2 inhibitor (AV-951, tivozanib) currently in Phase II clinical trials^23, 24^. This therapeutic strategy has been shown in numerous models to alter the tumor vasculature and reduce perfusion^25, 26^. As expected, there was a decrease in the *K*_trans_ values in the AV-951 only treated animals when compared with control; a result congruent with previously published data (compare Fig. 5c (pre-treatment) to Supplementary Fig. 8c (pre-treatment)). Although *K*_trans_ values between AV-951 and the combination hornerin siRNA and inhibitor group were not significantly different, there is a trend toward a further decrease in *K*_trans_ value for the combination therapy of AV-951 and *Hrnr* siRNA (Supplementary Fig. 8c). These data confirm that knockdown by *Hrnr* siRNA treatment results in reduction of both structural and functional parameters of tumor vasculature as well as reduced tumor outgrowth.

### Hornerin knockdown and VEGFR inhibitor combined treatment

**Fig. 6:**
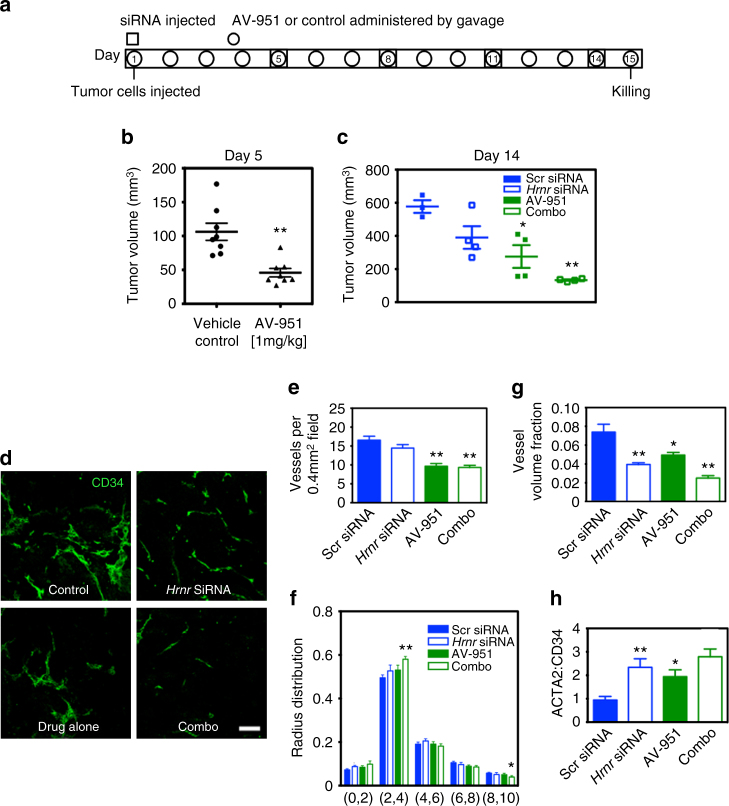
Hornerin knockdown in combination with VEGFR inhibition (AV-951) resulted in an enhanced reduction in tumor burden compared to monotherapy. **a** Timeline showing points of treatment over the course of the 15-day experiment. On day 5, control and 1.0 mg/kg AV-951-treated tumors were randomly selected to receive either Scr or *Hrnr* siRNA, resulting in four treatment groups. **b** Tumor volume (*N* = 8 tumors/treatment group) on day 5 post implantation in control and AV-951-treated mice. **c** Tumor volume was again calculated at day 14 post implantation. *N* = 4 in each treatment group, except control (*N* = 3). See Methods section for explanation of outlier exclusion. **d** Representative whole-mounted Z-stacked images of CD34^+^ vessels from each treatment group. **e** The number of CD34^+^ vessels per 0.4 mm^2^ field was quantified in 20 representative 5 μm sections (four tumors in each treatment group, five images per tumor). Images from 12 whole-mounted Z-stacks (50 μm sections) from each group (three fields per tumor, four tumors in each group) were analyzed by RAVE for **f** radius distribution and **g** vessel volume fraction. **h** Ratiometric quantification, determined by dividing ACTA2-positive pixels by CD34-positive pixels, of 12 images from each group (four tumors per group, three sections per tumor). *Scale bar* = 100 μm. Graphs represent mean ± SEM. *P < 0.05, **P < 0.005 by unpaired two-tailed *t*-test compared to control group

As we were able to decrease tumor size, vessel parameters, and vessel function with targeted hornerin knockdown, we investigated if the combination of this approach with VEGFR inhibition would produce an enhanced therapeutic response. The treatment schedule used in our study is outlined in Fig. 6a. As previously shown^24^, AV-951-treated mice exhibited tumor volumes that were decreased 2.3-fold compared to control-treated animals (Fig. 6b). Notably, treatment with *Hrnr* siRNA and AV-951 (combo) resulted in a 4.3-fold decrease in volume compared to the control group, indicating that inhibition of VEGF combined with hornerin knockdown results in an additive reduction in tumor growth (Fig. 6c). Notably, we observed that the most dramatic effect of hornerin knockdown occurred at the later stages of tumor outgrowth (days 11–14) (Supplementary Fig. 9).

We next wanted to determine if the reduced tumor burden observed in the monotherapy and combo groups was perhaps attributable to a lower degree of cell proliferation and/or enhanced cell death. To address this, expression of Ki67, a proliferation marker, and cleaved caspase 3, a marker of cellular apoptosis, were analyzed in tumor sections by immunofluorescent microscopy. Our results indicate that neither hornerin knockdown nor VEGFR inhibition altered overall tumor proliferation in either of the three treatment groups compared to control (Supplementary Fig. 10a, b). Interestingly, we did observe a two-fold increase relative to control in cleaved caspase 3 levels in tumors that had been treated with *Hrnr* siRNA or AV-951 alone, suggesting that enhanced apoptosis in the tumor may be responsible for the decreased tumor burden we observed in these two groups (Supplementary Fig. 10c, d). No significant difference from control was observed in tumors with combination treatment. Further analysis of the tumor cellularity indicated that the proportion of leukocytes (CD45^+^CD31^−^ cells) was not altered with hornerin knockdown, suggesting that immune cell infiltration into the tumor was not hornerin dependent (Supplementary Fig. 6a, c). Additionally, there were no observable differences in metastases between the groups.

The vessels from each treatment group were morphologically distinct (Fig. 6d). Control tumors had typical tumor vasculature—numerous, large, irregular vessels. As expected, *Hrnr* siRNA treatment resulted in vessels with smaller radii and tortuosity, but no change in the number of vessels per field (Fig. 6e–g). As previously published at this concentration^24^, vessels from AV-951 alone treated tumor-bearing animals were morphologically similar to control vessels; however, fewer vessels were observed. The combination therapy of *Hrnr* siRNA and AV-951 resulted in fewer vessels in addition to morphologic differences in the vessels. Quantitation with RAVE revealed the presence of an overall shift to smaller radii and VVF in the combo group compared to control, and a trend toward increased pericyte coverage (Fig. 6h).

**Fig. 7:**
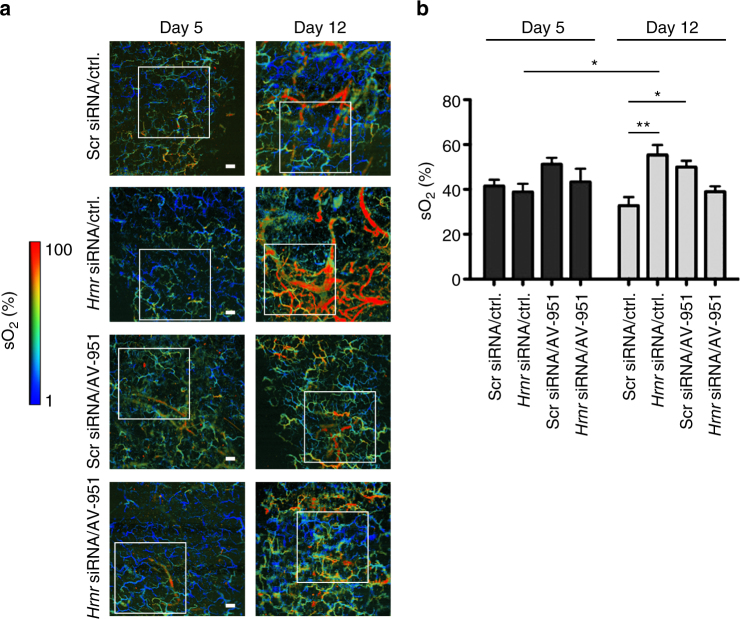
*Hrnr* siRNA and VEGFR inhibitor-treated mice display elevated hemoglobin oxygen saturation relative to control Scr siRNA mice. **a** Mice were inoculated subcutaneously with 5.0 × 10^5^ L3.6pl cells in Matrigel/HBSS and treated once daily by oral gavage with VEGFR inhibitor (rows 3 and 4) or vehicle ctrl. (rows 1 and 2) starting at day 1 post injection. Scr siRNA (rows 1 and 3) or *Hrnr* siRNA (rows 2 and 4) solutions were injected into the tumor at day 6 and day 9. Representative images of tumors from each of the four treatment groups at day 5 and day 12 display the blood oxygen saturation levels. A region of interest (ROI; *white box*) of equivalent dimensions (area) was defined and then applied in a blinded manner to each image. **b** Blood oxygen saturation levels were determined for each treatment group by analyzing the ROIs from four tumors/group. *Scale bar* = 200 µm. Graphs represent mean ± SEM. *P ≤ 0.05, **P ≤ 0.01 by unpaired two-tailed *t*-test

We further evaluated hornerin regulation of tumor vasculature through photoacoustic microscopy (PAM), which permitted non-invasive analysis of critical vessel parameters such as blood flow rate, hemoglobin, and oxygen saturation in the same tumor over the course of treatment. Our data revealed that prior to hornerin knockdown and 4 days post initiation of AV-951 treatment, there was no difference in either tumor blood hemoglobin or oxygen saturation as determined by PAM (Fig. 7a, b (day 5). However, while hemoglobin levels remained unchanged in tumors following hornerin knockdown and continued VEGFR inhibition, we did observe that oxygen saturation was elevated in the *Hrnr* siRNA (1.7-fold) and AV-951 (1.5-fold) cohorts compared to control (Fig. 7a, b (day 12)). This difference was notably ablated in the combination treatment group. These data indicate that in our model, hornerin knockdown and VEGFR inhibition alone function to increase tumor blood oxygen levels, and provide additional evidence that critical parameters of tumor vasculature are modulated by hornerin.

## Discussion

Despite preclinical and clinical successes, anti-angiogenic therapies, many of which focus on VEGF inhibition, have been hampered by acquired resistance mechanisms. We hypothesized that redundant signaling pathways exist in the tumor vasculature that circumvent monotherapies, and that the identification of VEGF-independent regulatory proteins may provide targets for dual anti-angiogenic treatment modalities. To identify these potential targets, we utilized phage display screening and functional proteomics methods, primarily because of its enormous potential to find unbiased targets with no a priori knowledge. Functionalizing the phage for use as a proteomics tool has so far identified novel proteins in PDAC such as pyruvate kinase M2 and plectin—both of which are normally cytoplasmic but are secreted and cell surface associated in cancer, similar to our findings with hornerin^16^. Of the identified proteins, plectin is the most studied and developed biomarker for PDAC and its importance in tumor biology was recently illuminated^27^. Since the original discovery as a PDAC biomarker, plectin has been published as being expressed in other diseases such as bladder^28^, head and neck squamous cell carcinoma^29^, and esophageal cancer^30^, indicating that our phage-based functional proteomics methods are robust and can lead to the identification of proteins of importance. After three rounds of in vivo phage screening, validation in vitro, and phage-based functional proteomics, we identified hornerin as a non-VEGF upregulated protein differentially expressed in tumor vessels. From this screen, we also identified other potentially interesting peptides and are working to identify their binding partners.

The identification of elevated hornerin expression in this setting is novel and contributes significantly to the relatively small volume of literature on hornerin regulation and function. Hornerin is a member of the S100 family of proteins, a group of calcium binding proteins involved in the maintenance of calcium homeostasis, as well other fundamental cellular processes and signaling cascades^31–33^. Several family members have been implicated in a variety of cancers^34, 35^, and significant attention has been directed to their role in receptor for advanced glycation end products activation^36–38^. The initial hornerin studies focused on expression in the cornified epidermal layer and regulation in chronically psoriatic skin^12, 13, 39^. Subsequent, but limited, investigation has addressed the intersection of hornerin with cancer, as highlighted in a recent report by Fleming et al.^40^ that showed increased hornerin expression in the development of murine mammary glands and in breast tumor cells associated with high tumorigenicity. Hornerin had not been evaluated in endothelial cells prior to this study, and our identification of hornerin specifically on the endothelium adjacent to pancreatic tumor outgrowth directed us to investigate its function under conditions found in a growing tumor.

The in vivo reduction of endothelial hornerin expression by intratumoral siRNA injection resulted in tumors with vessels that were morphologically distinct—characterized by smaller radii and reduced tortuosity. Further, we measured tumor vessel function using DCE-MRI, a non-invasive tool to track tumor vessel development^24, 41, 42^ and investigate vascularity and perfusion. Our results show decreased Gd-DTPA uptake and decreased *K*_trans_ value in tumors treated with *Hrnr* siRNA, suggesting that hornerin knockdown results in both structural and functional vascular changes that lead to decreased tumor growth. Further, tumors with decreased hornerin expression had higher oxygen saturation levels. Elevated tumor blood oxygen is a characteristic of patients who display improved prognosis and survival following anti-angiogenic and other treatment modalities, however this does not appear to be a universal phenomenon^43–45^. Other reports indicate that newly diagnosed glioblastoma patients who are responsive to the VEGFR kinase inhibitor cediranib display improved perfusion and oxygen delivery to the tumor, and also highlight the application of advanced imaging techniques to better understand the interplay of tumor oxygen status with tumor growth and response to therapy^46, 47^. Our data showing that hornerin alters tumor perfusion and blood oxygen saturation suggest that it may be involved mechanistically at several regulatory nodes that govern tumor oxygen status, a feature that is currently being investigated in our lab.

Finally, our combination therapy results coincide with an emerging body of evidence that suggests that compensatory pathways could additionally be responsible for non-durable clinical results when targeting VEGF^48^. Anti-vascular therapies, which are almost always synonymous with VEGF inhibitors, have achieved success pre-clinically and clinically and have opened new avenues for anti-tumor therapy^3, 4^. However, the durable patient response and efficacy have been marginal, measuring increased patient survival in months. Of the tumors that respond, most recur and are no longer responsive to anti-VEGF therapy^49^. One hypothesis could be the presence of compensatory or redundant pathways, which are well established in tumor therapy research. Consequently, clinical trials devoted to synergistic combinations of drugs have proliferated^7, 10^, although most are aimed at pathways present in the cancer cells. Compensatory pathways involving the VEGF and fibroblast growth factor (FGF) pathways have been described in tumor vessel studies^50^. An approach involving both the VEGF pathway and another important pathway could provide increased positive therapeutic outcomes. The existence of VEGF-independent compensatory pathways is an emerging concept that has been investigated by, among others, the Hanahan group. Recent evidence suggests that activation of the FGF2 pathway could constitute a potential resistance mechanism to anti-VEGF therapy^50^. When FGF2 signaling was blocked by FGF2 trap, resistance to the anti-VEGFR2 antibody DC101 was eliminated^50^. In further studies by the same group, brivanib, a dual VEGF/FGF inhibitor, suppressed anti-VEGF (DC101 and sorafenib) -acquired resistance^51^.

To expand on this concept, we asked whether decreasing hornerin protein levels in combination with VEGFR2 inhibition would produce synergistic or additive reductions in tumor volume. Each monotherapy, hornerin knockdown and VEGFR inhibition, produced a two-fold reduction in tumor volume. When the therapies were combined, the resulting decrease in tumor volume was four-fold. Interestingly, tumor-cleaved caspase three levels were elevated with either treatment alone but not in mice subjected to combination therapy, suggesting that perhaps hornerin knockdown and VEGFR inhibition synergize through a yet unidentified mechanism to induce stasis of tumor growth during the early stages of progression. The identification of hornerin and its additive effects in combination with VEGF inhibition represents a promising avenue for development of targeted therapeutics against hornerin.

The implication of hornerin in a VEGF-independent signaling cascade that modulates tumor vessel parameters prompts a need to discover the factors present in the L3.6pl secretome that were responsible for hornerin upregulation. Knowledge of the signaling pathways that govern hornerin expression in any biological context, however, remains limited, with calcium indicated as one of the few regulatory molecules in the epidermis^12, 14^. Knowledge of expression modulators could direct further studies into the signaling and mechanism of hornerin in tumor angiogenesis. To assess the potential for growth factor-mediated hornerin upregulation, we first determined the highest expressed non-VEGF growth factors in the TCM and treated HUVECs with these factors at published B-max concentrations (Supplementary Fig. 11a, b). Our preliminary results suggest that interleukin 12 (IL-12) and basic FGF (bFGF) may stimulate hornerin expression. It is also established that the L3.6pl cell line produces relatively high levels of epidermal growth factor (EGF) and that in tumor models signaling through the EGF receptor potentiates endothelial cell survival^52^. We also observed that hornerin expression increases in HUVECs in response to EGF stimulation, suggesting that one potential mechanism of elevated hornerin in the tumor endothelium is due to tumor cell-derived EGF, however these results remain to be explored in the tumor setting (Supplementary Fig. 11c). Interestingly, it appears that the response to EGF stimulation may be wide ranging, as we observed a concentration-dependent increase in expression to EGF treatment in L3.6pl cells and a more modest response in keratinocytes (Supplementary Fig. 11d, e). Elucidation of the factors, those that regulate hornerin and others that function downstream of hornerin, could have substantial clinical relevance by opening additional avenues of therapeutic intervention targeting the tumor vasculature.

While our study does reveal a critical role for endothelial cell-hornerin in regulating vessel parameters that are characteristic of the tumor vasculature, it does not address the function of hornerin in other cell types in the tumor microenvironment. Future work utilizing conditional knockdown models of hornerin in different cell lineages could provide additional clarity. Preliminary evidence presented in this study suggests that hornerin knockdown does not alter accumulation of leukocytes in the tumor microenvironment. It should not be overlooked that our current understanding attached to hornerin expression, regulation, and function has developed on a foundation laid by other closely related S100 fused-type protein family members, however very little experimental data exist to validate these assumptions. The fact that hornerin is expressed and functional in the novel biological context revealed in our study suggests that much remains to be learned about hornerin at the fundamental level.

## Methods

### Animals

All experiments were performed on male 9–10-week-old athymic nude (nu/nu) mice purchased from Charles River Laboratories (Wilmington, MA, USA). All procedures were approved by the University of Virginia Institutional Animal Care and Use Committee and in compliance with federal regulations.

### Study design

A tumor sample size of four was determined by power analysis based on prior tumor xenograft studies. For our 80% power analysis, the difference between means was 200 mm^3^, standard deviation was 100 mm^3^, and *P*-value < 0.05 (*P* < 0.05). Tumors were measured at least twice weekly until their volume was >1000 mm^3^, at which point animals were killed. Tumor size data were collected from each tumor, with the exception of one tumor in the combination treatment experiment (Fig. 6) that was identified with 90% confidence via Grubb’s Outlier Test. The following number of mice in each treatment cohort was evaluated for tumor outgrowth: (Scr siRNA/ctrl., *N* = 15; *Hrnr* siRNA/ctrl., *N* = 14; Scr siRNA/AV-951, *N* = 8; *Hrnr* siRNA/AV-951, *N* = 8).

### Phage screen and sequencing

To conduct the phage screen, 10 µL of PhD-7 phage display library (New England Biolabs, Ipswich, MA, USA) was combined with 190 µL of Dulbecco’s phosphate buffered saline (DPBS) and injected intravenously into mice harboring orthotopic xenograft PDAC tumors. Phage circulated for 8–10 min before the animals were killed and cardiac perfused with 30 mL of DPBS. Tumors were minced into 1–2 mm segments and Dounce homogenized in a solution consisting of DPBS + 1% TritonX, ethylene diamine tetraacetic acid, and protease inhibitors (Thermo Scientific, Waltham, MA, USA). The phage was amplified according to manufacturer’s instructions and two additional rounds of screening were completed as described above.

After three rounds of screening, phage were isolated from plaques and placed in water, upon which 10 µL of bacterial lysis/phage water solution was added as template for PCR amplification. Primer sequences:

FWD: 5′-CCTTTAGTGGTACCTTTCTAT-3′

REV: 5′-GCCCTCATAGTTAGCGTAACG-3′.

The amplicon was sequenced using a 3730 DNA Analyzer (Applied Biosystems, Foster City, CA, USA).

### Cell culture

L3.6pl tumor cells were obtained from Dr Craig Logsdon (University of Texas, MD Anderson Cancer Center, Houston, TX, USA), grown in minimal essential medium (MEM) with 10% fetal bovine serum and penicillin/streptomycin, and passaged every 72 h. All experiments involving L3.6pl cells were completed from passage 10–20. HUVEC were purchased from Lonza (Basel, Switzerland), grown in endothelial growth media supplemented with an endothelial cell SingleQuot kit (Lonza), and characterized as >90% double-positive for CD31/CD105 at passage four. HUVEC were passaged every 72 h and used between passages two through eight. Human primary epidermal keratinocytes (American Type Culture Collection (ATCC), Manassas, VA, USA) and COCA mouse epidermal keratinocytes (Sigma Aldrich, St. Louis, MO, USA) were cultured in dermal cell basal media supplemented with a keratinocyte growth kit (ATCC). All cell lines were tested for mycoplasma contamination and maintained in a humidified atmosphere containing 5% CO_2_ at 37 °C.

### Preparation of tumor-conditioned media

Tumor-conditioned media (TCM) were prepared from L3.6pl -conditioned media that had been collected after 72 h of production and passed through a cell culture 0.22 μm syringe filter (Argos Technologies, Elgin, IL, USA). Media were concentrated to 10× using Ultra-10 10 kilodalton molecular weight cutoff centrifugation filter units (Millipore, Darmstadt, Germany) and stored at −20 °C.

### Phage-based ELISA

HUVEC were plated in MaxiSorp 96-well plates (NUNC, Rochester, NY, USA) and cultured for 24 h, at which time the cells were treated with media containing VEGF (VEGFCM) at B-max concentration^53^ or TCM for an additional 24 h. ELISA was initiated by adding 10^10^ isolated phage to each well for a 1 h incubation. After three washes with DPBS + 0.1% Tween-20 (DPBST), the cells were incubated with horse radish peroxidase (HRP)-tagged anti-M13 antibody (GE Healthcare, Little Chalfont, UK, 27-9421-01; (1:3000)) for 30 min. The plates were washed three additional times (DBPST) then developed with 200 µL 3,3′,5,5′-Tetramethylbenzidine (Sigma) for 10 min. The A_650_ was measured on a microplate reader (Molecular Devices, Sunnyvale, CA, USA).

### Two-way ANOVA analysis

Absorbance values were corrected by comparison to unmodified control KE phage. To validate and confirm which clones reliably bound TCM and/or VEGFCM over VC, two-way ANOVA analysis (via MATLAB software) was completed on data from two separate experiments (*N* = 6). Independent variables tested were conditioned media type and the two sets of experimental data for each day. Also, an interaction term was computed. For clones to be classified as a TCM or both TCM and VEGFCM binder, a significant difference (*P* < 0.01) had to be achieved in the conditioned media vs. VC comparison, but not between experimental data sets or the interaction factor.

### FITC labeling of phage clones

Following an established protocol described briefly here^12^, phage were first suspended at a concentration of 10^9^ plaque forming units (pfu)/µL in DPBS. FITC was solubilized in dimethyl sulfoxide (25 µg/2 µL) and added to 200 µL of the phage solution. Following a 1 h incubation at RT with rocking, the labeled phage were purified by three rounds of polyethylene glycol precipitation and resuspended in DPBS. FITC-labeled phage (200 µL) was injected via tail vein and allowed to circulate for 4 h. The animal was cardiac perfused prior to removal of the tumored pancreas for cryopreservation.

### Immunoblot

HUVECs were cultured in 10 cm dishes for 24 h prior to a change to media containing VEGF (at B-max concentration), TCM, or VC. After 24 h treatment, the cells were washed with DPBST two times and lysed with radioimmunoprecipitation assay (RIPA) buffer (Thermo Scientific). Lysate protein concentrations were measured by the bicinchoninic acid assay (Pierce, Waltham, MA, USA) and equivalent amounts of protein were loaded into precast 4–15% tris-glycine eXtended (TGX) polyacrylamide gels (Bio-Rad, Hercules, CA, USA). The proteins were resolved by electrophoresis and transferred to nitrocellulose membranes using the iBlot system (Invitrogen, Carlsbad, CA, USA). The membranes were then washed in Tris-buffered saline + 1% Tween-20 (TBST), blocked for 30 min in TBST + 2% milk solution, and incubated overnight at 4 °C with primary antibodies anti-hornerin (Abcam, Cambridge, UK, ab78909; (1:1000)) and anti-HSP90 (Cell Signaling Technology, Danvers, MA, USA, 4877; (1:1000)). The following day the membranes were washed and subsequently incubated with the secondary antibodies anti-goat HRP (R&D Systems, Minneapolis, MN, USA, HAF109; (1:5000)) and anti-rabbit HRP (GE Healthcare, NA934V; (1:5000)) for 45 min at RT. After washing, the membranes were incubated in luminol/peroxide substrate reagents (Millipore), exposed to HyBlot autoradiography film (Denville Scientific, South Plainfield, NJ, USA), and developed. The films were scanned using a tabletop office scanner and converted to JPEG files for densitometry. Relative densitometry was computed using the “Gel Tool” in Image J (National Institutes of Health (NIH), Bethesda, MD, USA). For immunoblot detection of protein expression following EGF treatment, HUVECs were plated at a density of 0.15 × 10^6^ cells/well in six-well cluster plates in complete media. The adherent cells were subsequently washed twice with serum-free media and replenished with EGF-free complete media for 14 h, upon which the cells treated with EGF-free complete media supplemented with increasing concentrations of recombinant human EGF (PeproTech, Rocky Hill, NJ, USA). Lysate preparations were generated after 24 h treatment. L3.6pl cells were plated in complete media in six-well cluster plates at a density of 0.2 × 10^6^ cells/well. On day 2, the cells were replenished with serum-free media and serum starved overnight, upon which EGF was added at a concentration of 1, 10, or 100 ng/mL. Lysate preparations were generated after 48 h treatment. Human keratinocytes were plated at a density of 0.1 × 10^6^ cells/well in six-well plates, treated on day 2 with media containing either EGF or 2 mM CaCl_2_, and lysed after 48 h treatment. Lysate preparations were subjected to immunoblot using the primary antibodies anti-hornerin (Abcam; (1:1000)) and anti-beta actin (Cell Signaling, 3700, clone 8H10D10; (1:4000)), and corresponding secondary antibodies anti-goat HRP (R&D Systems; (1:10,000)) and anti-mouse HRP (R&D Systems, HAF018; (1:10,000)).

### Angiogenesis array

Major constituents of TCM were determined using a Human Angiogenesis Antibody Array (Affymetrix, Santa Clara, CA, USA) per manufacturer’s instructions. TCM and VC media were incubated on separate test strips and then assayed for elevated growth factors in TCM relative to VC. The film was developed, scanned, and subjected to densitometry analysis using Image J. The absolute difference between TCM and VC was computed. To obtain lysates for western blots, HUVEC cells were seeded in 10 cm circular dishes and allowed to grow for 48 h. Next, growth factors were added in a change of media at published B-max concentrations (IL-6^54^, IL-8^55^, IL-12^56^, TNF^57^, bFGF^58^) and the cells were incubated for an additional 24 h. The cells were then washed with DPBST two times and lysates were generated for immunoblot detection.

### De-identified human PDAC specimens

De-identified resected human PDAC specimens were obtained through the UVA Biorepository and Tissue Research Facility (BTRF) in accordance with the policies established by the University of Virginia Institutional Review Board for Health Sciences Research. Ten formalin-fixed, paraffin-embedded tumor samples were cut into 5 μm sections and analyzed for hornerin expression by immunohistochemistry. To accomplish this, the sections were deparaffinized with xylene and ethanol, treated with low pH antigen unmasking solution (Vector Labs, Burlingame, CA, USA) in a microwave for 20 min, and washed twice with DPBS. Next, endogenous peroxidase activity was quenched using 0.5% H_2_O_2_ in methanol for 30 min at room temperature. The slides were then washed, blocked for 30 min in DPBS + 5% BSA (blocking buffer), and incubated with anti-hornerin antibody (Sigma, HPA031469; (1:100)) in blocking buffer at 4 °C overnight. After three washes with DPBS, the slides were incubated with anti-rabbit HRP-labeled secondary antibody (GE Healthcare; (1:2500)) in blocking buffer for 30 min at RT. The signal was developed using 3,3′-diaminobenzidine (DAB) tablets (Thermo Fisher Scientific) and subsequently counterstained with hematoxylin (Thermo Fisher Scientific). The sections were then dehydrated in 100% ethanol (three washes), 100% xylene (two washes), and mounted with CytoSeal 60 (Thermo Scientific).

A certified pathologist (C.M.) scored the stained slides by stain intensity and percentage of positive cells. Endothelial cells were identified by a combination of cell morphology, nucleus shape, and presence of erythrocytes. Histological images were taken with an Olympus BX-41 microscope and QImaging Retiga-2000R camera.

### Tumor studies

For tumor implantation, L3.6pl cells were grown under normal culture conditions for 72 h prior to being trypsinized and enumerated. The cells were suspended in aliquots of 500,000 cells/50 µL Hanks Balanced Salt Solution (HBSS) (Corning, Manassas, VA, USA) and 50 μL of Matrigel (BD Biosciences, San Jose, CA, USA) was added to each aliquot of cells prior to implantation. The cell-Matrigel slurry was injected subcutaneously into nu/nu mice on the flanks or orthotopically into the pancreas^18^. The injection site for subcutaneous tumors was monitored daily for growth and tumor volume was calculated form caliper measurements using the formula (width^2^ × length)/2.

The VEGFR inhibitor AV-951 (Selleck Chemicals, Houston, TX, USA) was suspended in 0.5% methocel + 0.5% Tween-20 in HBSS to increase viscosity and 50 µL of 1.0 mg/kg AV-951 or VC solution was administered once daily by oral gavage (22-gauge gavage needle (Kent Scientific, Torrington, CT, USA) connected to a 1 mL syringe).

Silencer Select mouse hornerin siRNA (*Hrnr* siRNA; 5′-GCAUGGAUCUUGUUGCGGUtt-3′) (Invitrogen) was aligned against human hornerin transcripts using the basic local alignment tool (BLAST) algorithm and no match was returned. To prepare *Hrnr* siRNA and Silencer Select Negative Control #1 Scr siRNA (Invitrogen; catalog number 4390844) for injection, 10 µg of hornerin or control siRNA were combined with in vivo-jetPEI transfection reagent (Polyplus, France) in solution with 5% glucose per Polyplus instructions (total volume 50 µL). After a 30 min. incubation, the siRNA solutions were injected slowly into the center of the tumor. A second independent hornerin knockdown experiment was conducted using a pooled set of three unique mouse *Hrnr* siRNA duplexes (5′-GGAAUUAAGUUAGGAAGAAUAAUTT-3′; 5′-AGAGUGCAUAGACAAGUAGAAACCT-3′; 5′-CCCUACUUCAGAACAAUAUGGGUCT-3′) and Scr siRNA (Origene, Rockville, MD, USA). The reagent preparation and injection protocol were similar to that described above.

Following completion of the treatment schedule, the mice were killed, cardiac perfused, and the tumors were excised and fixed in formalin overnight. The tumors were paraffin embedded and cut into 5 (thin) or 50 µm (thick) sections. Tumor vessel parameters were evaluated in the following number of tumors for each treatment cohort: (Scr siRNA/ctrl., *N* = 10; *Hrnr* siRNA/ctrl., *N* = 10; Scr siRNA/AV-951, *N* = 4; *Hrnr* siRNA/AV-951, *N* = 4).

### Immunofluorescent staining

Tumor Sections were deparaffinized, rehydrated, heated in antigen retrieval solution (Vector), blocked for 30 min in blocking buffer, and incubated with anti-CD34 antibody (Abcam, ab8158, clone MEC14.7; (1:100)) in blocking buffer overnight at 4 °C. The sections were then incubated with goat anti-rat FITC secondary antibody (Abcam, ab6840; (1:100)) in blocking buffer prior to mounting with Prolong Gold Antifade reagent (Invitrogen). Anti-actin, α-smooth muscle Cy3 antibody (Sigma, C6198, clone 1A4; (1:200)) was added to the primary antibody solution if assaying for alpha-smooth muscle actin. To compare hornerin expression in vessels and tumor tissue treated with Hrnr siRNA and Scr siRNA, thin paraffin sections were deparaffinized and stained with anti-CD34 and anti-hornerin antibodies described. Images were collected using a Nikon TE 2000-E2 microscope (Nikon Instruments) equipped with a Melles Griot Argon Laser System (Melles Griot, Carlsbad, CA, USA) and a Nikon D-Eclipse C1 confocal attachment and analyzed by Measurement Tool in Image J. To measure the relative hornerin expression in tumor vessels, three vessel segments per image were isolated and their mean intensity and area were measured and recorded. A weighted mean intensity was calculated to correct for differences in selected area. To measure the relative hornerin expression in tumor tissue, the mean intensity from three square sections (75 × 75 pixel) of tumor tissue per image was measured and recorded.

Vessel parameters were measured using RAVE software^18^. For detection of proliferation and apoptosis, the sections were incubated with the primary antibodies anti-Ki67 (Abcam, ab15580; (1:200)) or anti-cleaved caspase 3 (Cell Signaling, 9661; (1:200)), respectively, overnight at 4 °C followed by incubation with anti-rabbit AF594 (Abcam, 150080; (1:200)).

Human keratinocytes or COCA cells were plated on fibronectin-coated coverslips and treated with 2 mM CaCl_2_ to induce differentiation. After 48 h. the cells were fixed in 4% paraformaldehyde, permeabilized in methanol, blocked in 5% BSA/PBS, and incubated with anti-hornerin antibody (Sigma; (1:150)) and anti-beta actin FITC (Sigma, F3022, clone AC-15; (1:250)). Following incubation with anti-rabbit AF594 (1:400) the coverslips were mounted in Prolong Gold + DAPI and analyzed by microscopy. The acquired images were prepared using Image J.

### Quantitative PCR

Day 10 tumors were injected with either Scr siRNA or *Hrnr* siRNA and excised 20 h. later. The tumors were immediately washed in PBS (+Mg/+Ca), minced with scalpels, treated with Collagenase Type II (Worthington Biochemical Corporation, Lakewood, NJ, USA; (2.0 mg/mL) serum-free DMEM, 100 min at 37 °C), and filtered through a 40 μm cell strainer. The cell suspension (filtrate) was incubated with the following antibody/dye panel for FACS; anti-CD45 AF488 (1:100) (Biolegend, San Diego, CA, USA, 103121, clone 30-F11), anti-CD31 AF647 (1:100) (Biolegend, 102415, clone 390), LIVE/DEAD violet stain (1:200) (Thermo Fisher). Flow cytometry analysis was completed on the Cyan ADP LX (Beckman Coulter). Compensation and data analysis was completed using FlowJo software (Ashland, Oregon). FACS was completed in the UVA flow cytometry core on the Influx cell sorter (BD Biosciences, Franklin Lakes, NJ, USA).

RNA was isolated from FACS-sorted CD31^+^CD45^−^ cells according to the manufacturer’s protocol (Qiagen mini-prep kit), and concentration and purity were determined using a NanoDrop Spectrophotometer (Thermo Fisher). Complimentary DNA (cDNA) was generated (Qiagen QuantiTect Reverse Transcription Kit) and subjected to pre-amplification (SsoAdvanced PreAmp Supermix, Bio-Rad). For this reaction, 50 nM of each primer, mouse cyclophilin B (Forward 5′-TGCCGGAGTCGACAATGAT-3′; Reverse 5′-TGGAGAGCACCAAGACAGACA-3′) and mouse hornerin (Forward 5′-CCTGGAAAGCATTGTCACTGT-3′; Reverse 5′-CGGTGTCTGGATCATCTGG-3′)^59^ were combined with the PreAmp Supermix and cDNA template, and subjected to the following thermal cycling protocol: 3 min. at 95 °C (polymerase activation and DNA denaturation), followed by 20 cycles of 15 s at 95 °C (denaturation) and 4 min. at 58 °C (annealing/extension). The pre-amplified cDNA was subjected to qPCR (Qiagen SYBR Green PCR kit; 60 °C annealing temperature, 60 cycles). Melt curve analysis was performed on the hornerin and cyclophilin primer sets prior to data collection and normalized gene expression was determined by the delta delta Ct method.

### DCE-MRI and *K*_trans_ measurement

To assess the in vivo function of tumor vascularity, DCE-MRI was performed on a 7.0T Clinscan small animal imaging system (Bruker, Ettlingen, Germany) using a 30 mm inner-diameter birdcage radiofrequency coil. Mice (*N* = 7) were imaged prior to treating tumors with *Hrnr* and Scr siRNA and again 6 days after treatment. Mice treated with the VEGFR inhibitor AV-951 (*N* = 5) were image pre-siRNA treatment and 6 days post-treatment. Mice were anesthetized with 1.25% isoflurane anesthesia, which was maintained during imaging. Respiration and ECG were monitored during imaging using an MRI—compatible system (SA Instruments, Stony Brook, NY, USA). An indwelling tail vein catheter was established for administration of the gadolinium contrast agent Gd-DTPA, which is required in DCE imaging. Mice were placed supine in the imaging system, and body temperature was maintained at 36 °C ± 1 °C using circulating, temperature controlled water. Tumors were localized using a spin-echo imaging sequence, and an imaging plane that transected both tumors was chosen for DCE imaging.

A dual-Gd-bolus DCE-MRI acquisition strategy^60^ was employed, which uses a low dose of contrast agent to accurately acquire the undistorted Gd concentration vs. time curve in the blood pool and a higher dose of contrast agent to acquire the Gd concentration vs. time curve in the tumor with high signal-to-noise ratio, since Gd-DTPA is known to have signal saturation effects at high doses in the blood pool^60^. Short-axis ECG-gated images of the left ventricle were acquired using a saturation recovery gradient echo pulse sequence with the following imaging parameters: number of images = 500, 1 image per heartbeat, echo time/repetition time/saturation delay time = 0.8/1.6/10 ms, excitation flip angle = 25°, image resolution = 0.47 × 0.47 × 1 mm, field of view = 30 mm, Gd-DTPA dose = 0.025 mmol/kg, imaging time ~1 min. ECG and respiratory-gated images of the tumors were similarly acquired using a saturation recovery gradient echo pulse sequence with the following imaging parameters: number of images = 350, echo time/repetition time/saturation delay time = 1.1/2.0/200 ms, excitation flip angle = 25°, averages = 2, image resolution = 0.195 × 0.195 × 1.5 mm, field of view = 25 mm, Gd-DTPA dose = 0.15 mmol/kg, imaging time ~5 min. Gd-DTPA concentration vs. time curves were generated by drawing regions of interest around the left ventricular cavity and each tumor. Each blood pool Gd-DTPA concentration vs. time curve was fit with a gamma-variate function during Gd wash-in and extrapolated with an exponential decay function during Gd wash-out to have the same temporal duration as its corresponding tumor Gd concentration vs. time curves. Tracer kinetic Kety model analysis^61^ was applied to the left ventricular and tumor Gd concentration vs. time curves, which yielded an estimate of the volume transfer constant *K*_trans_, a widely-accepted MRI measurement of vascularity^62–64^.

### Photoacoustic microscopy

Using an established PAM system^65–67^, we characterized the tumor vasculature in vivo. Two laser wavelengths, 532 and 559 nm, were used to simultaneously quantify microvascular diameter, oxygen saturation, and blood flow at the same spatial scale^66^. The applied optical energies were 100 and 75 nJ, which are below the maximum permissible exposure energy. Throughout the experiment, mice were maintained under anesthesia with 1.5% vaporized isoflurane and the body temperature was kept at 37 °C. All experimental procedures were carried out in conformity with the laboratory animal protocol approved by the Animal Care and Use Committee at the University of Virginia.

### In vitro siRNA experiments

For in vitro siRNA-mediated hornerin knockdown, COCA cells and human keratinocytes were plated at a density of 0.1 × 10^6^ cells/well in a six-well cluster plate. The cells were treated the following day with Scr siRNA or *Hrnr* siRNA (Invitrogen; siRNA sequences identical to those utilized in tumor studies) following an established protocol described here^68^. Briefly, 100 µL of 10 µM siRNA and 12.5 µL of Lipofectamine 2000 (LF2000) (Invitrogen) were added to separate 625 µL OptiMEM (Thermo Fisher Scientific) aliquots. After 5 min at RT, the solutions were mixed and incubated for an additional 30 min, upon which 5 ml of keratinocyte media were added. The cells were incubated overnight in 3 ml of the siRNA/LF2000/media mixture, replenished the following morning with media containing 2 mM CaCl_2_ to induce differentiation, and lysed in RIPA buffer 8 h post-media change. Lysate preparations were subjected to immunoblot using the primary antibodies anti-human hornerin (Abcam; (1:1000)), anti-mouse hornerin (Santa Cruz Biotechnology, Dallas, TX, USA, sc164605; (1:200)), or anti-beta actin (Cell Signaling; (1:4000)), and respective secondary antibodies anti-goat HRP (R&D Systems; (1:10,000)), and anti-mouse HRP (R&D Systems; (1:10,000)). Uncropped hornerin immunoblots are represented in Supplementary Fig. 4.

### Mass spectrometry

The gel piece was transferred to a siliconized tube and washed and destained in 200 µL 50% methanol overnight. The gel pieces were dehydrated in acetonitrile, rehydrated in 30 µL of 10 mM dithiolthreitol in 0.1 M ammonium bicarbonate, and reduced at room temperature for 0.5 h. The DTT solution was removed and the sample alkylated in 30 µL 50 mM iodoacetamide in 0.1 M ammonium bicarbonate at room temperature for 0.5 h. The reagent was removed and the gel pieces dehydrated in 100 µL acetonitrile. The acetonitrile was removed and the gel pieces rehydrated in 100 µL 0.1 M ammonium bicarbonate. The pieces were dehydrated in 100 µL acetonitrile, the acetonitrile removed and the pieces completely dried by vacuum centrifugation. The gel pieces were rehydrated in 20 ng/µL trypsin in 50 mM ammonium bicarbonate on ice for 10 min. Any excess enzyme solution was removed and 20 µL 50 mM ammonium bicarbonate added. The sample was digested overnight at 37 °C and the peptides formed extracted from the polyacrylamide in two 30 µL aliquots of 50% acetonitrile/5% formic acid. These extracts were combined and evaporated to 15 µL for MS analysis.

The LC-MS system consisted of a Thermo Electron Velos Orbitrap ETD mass spectrometer system with a Protana nanospray ion source interfaced to a self-packed 8 cm × 75 µm id Phenomenex Jupiter 10 µm C18 reversed-phase capillary column. About 7.5 µL of the extract was injected and the peptides eluted from the column by an acetonitrile/0.1 M acetic acid gradient at a flow rate of 0.5 µL/min over 0.3 h. The nanospray ion source was operated at 2.5 kV. The digest was analyzed using the double play capability of the instrument acquiring full scan mass spectra (Orbitrap, 60 K resolution) to determine peptide molecular weights followed by product ion spectra (ion trap) to determine amino-acid sequence in sequential scans. Data collection was maximized by using default dynamic exclusion settings.

The data were analyzed by database searching using the Sequest search algorithm within Proteome Discoverer (v1.2) against IPI Human (v3.78, 86,702 entries). The peptides and proteins identified for the sample are displayed using Scaffold (v3.0.6) with the following settings (parent = 10 ppm, fragment = 1 Da, trypsin, 60% peptide threshold, 90% protein threshold, Xcorr > 1, 8, 2.2, 2.5, 3.5 for +1, +2, +3, +4, >C = 57 fixed, M = 167 variable). The proteins of particular interest are examined manually at the spectral level to determine if true positives.

### Statistics

Unless otherwise noted, all data are represented as mean ± SEM and all *P*-values were calculated using a two-tailed, unpaired student’s *t*-test. Two-way ANOVA statistical methods are described above.

### Data availability

The authors declare that all the data supporting the findings of this study are available within the article and its supplementary information files and from the corresponding author upon reasonable request.

## Electronic supplementary material

Supplementary Information

Supplementary Movie 1

Supplementary Data 1
