# Supplementary material for: Identification of the S100 fused-type protein hornerin as a regulator of tumor vascularity
Source: Nat Commun. 2017 Sep 15;8:552. doi: 10.1038/s41467-017-00488-6 (PMC5601918; doi:10.1038/s41467-017-00488-6)
Supplement: Supplementary file 1 — Supplementary Information [file 41467_2017_488_MOESM1_ESM.pdf]

Description of Supplementary Files

File Name: Supplementary Information
Description: Supplementary Figures and Supplementary Table

File Name: Supplementary Movie 1
Description: Scr and Hrnr siRNA treated tumors are demarcated by a dashed white line. GdDTPA was injected 4 seconds after start of playback.

File Name: Supplementary Data 1
Description: MS/MS data from the PTEM 9 pull-down.

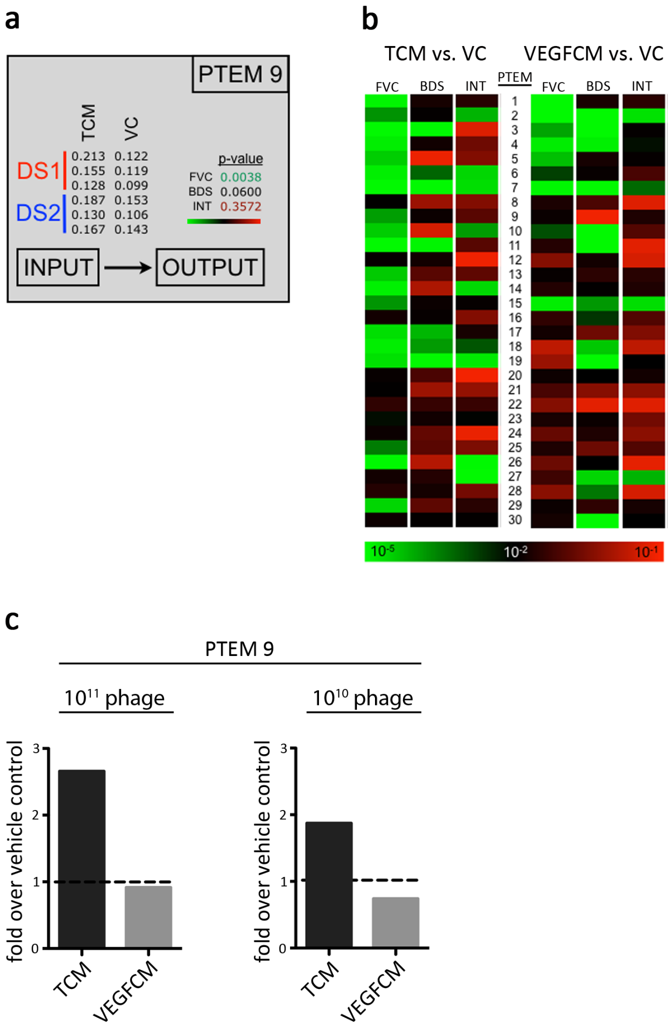
a
PTEM 9
TCM
VC
DS1
0.213 0.122
0.155 0.119
0.128 0.099
DS2
0.187 0.153
0.130 0.106
0.167 0.143
p-value
FVC 0.0038
BDS 0.0600
INT 0.3572
INPUT
OUTPUT
b
TCM vs. VC
VEGFCM vs. VC
FVC
BDS
INT
PTEM
FVC
BDS
INT
1
2
3
4
5
6
7
8
9
10
11
12
13
14
15
16
17
18
19
20
21
22
23
24
25
26
27
28
29
30
10^{-5}
10^{-2}
10^{-1}
c
PTEM 9
10^{11} phage
10^{10} phage
fold over vehicle control
0
1
2
3
TCM
VEGFCM

Supplementary Figure 1. ELISA and two-way ANOVA analysis identified clones that displayed specific binding to TCM treated HUVEC. (**a**) ELISA data from PTEM 9 is organized in matrix format, forming the input to two-way ANOVA analysis. Two independent experiments (DS1 and DS2; three replicates in each) were analyzed. Analysis yields three probabilities or P values: fold-over vehicle control (FVC), between data sets (BDS), and an interaction (INT). (**b**) Heatmap showing P values for the comparisons TCM versus VC (left) and VEGFCM versus VC (right). An intensity scale is located below the heatmap. (**c**) ELISA with PTEM 9 clone using two different phage concentrations. Graphs represent average PTEM 9 selective binding over M13KE control phage (no peptide displayed) under the three conditions VC, TCM, and VEGFCM, as determined by ELISA. Samples were run in triplicate for each condition.

a

b

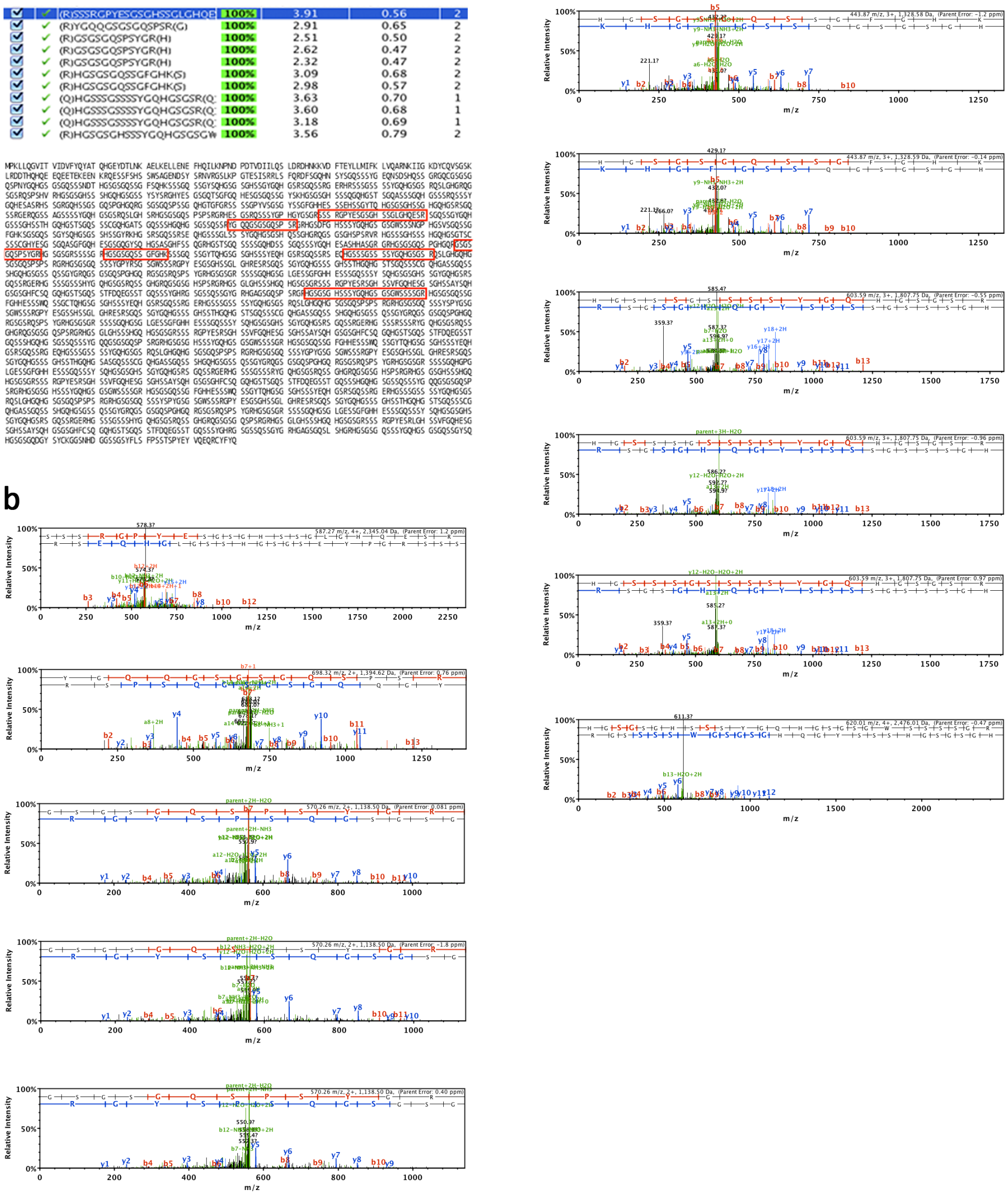

(R)SSSRGPYESGSGHSSGLGHQE 100% 3.91 0.56 2
(R)YGQQGSGSGQSPSR(G) 100% 2.91 0.65 2
(R)GSGSGQSPSYGR(H) 100% 2.51 0.50 2
(R)GSGSGQSPSYGR(H) 100% 2.62 0.47 2
(R)GSGSGQSPSYGR(H) 100% 2.32 0.47 2
(R)HGSGSGQSSGFGHK(S) 100% 3.09 0.68 2
(R)HGSGSGQSSGFGHK(S) 100% 2.98 0.57 2
(Q)HGSSSGSSSSYGQHGSGSR(Q 100% 3.63 0.70 1
(Q)HGSSSGSSSSYGQHGSGSR(Q 100% 3.60 0.68 1
(Q)HGSSSGSSSSYGQHGSGSR(Q 100% 3.18 0.69 1
(R)HGSGSGHSSSYGQHGSGSGW 100% 3.56 0.79 2
Relative Intensity
m/z

Supplementary Figure 2. PTEM 9 pulldown. (**a**) Sequences of the 11 identified peptides following MS/MS analysis of the gel band derived from the PTEM 9 pulldown assay. Sequences (boxed in red) were overlaid on the amino acid sequence of human hornerin. The percent coverage (MS/MS peptides/hornerin amino acid sequence) is 3.5%. (**b**) The spectra for each of the 11 identified peptides.

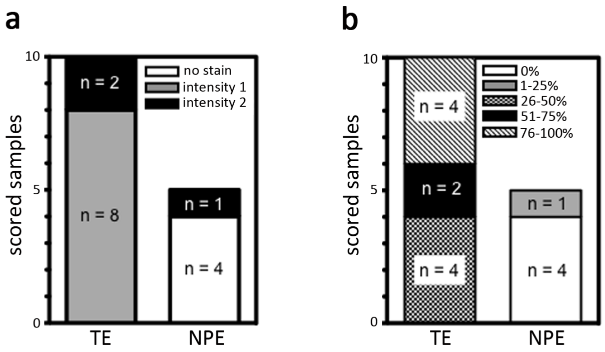
a
b
scored samples
10
5
0
TE
NPE
n = 2
n = 8
n = 1
n = 4
no stain
intensity 1
intensity 2
n = 4
n = 2
n = 4
n = 1
n = 4
0%
1-25%
26-50%
51-75%
76-100%

Supplementary Figure 3. Comparative hornerin expression in human pancreas specimens. The tumor endothelium (TE) and normal pancreas endothelium (NPE) in 10 human PDAC specimens were scored for hornerin expression based on (**a**) immunohostochemical staining intensity and (**b**) the percentage of positively stained cells. N refers to the number of specimens that fit the specific criteria for each method of analysis.

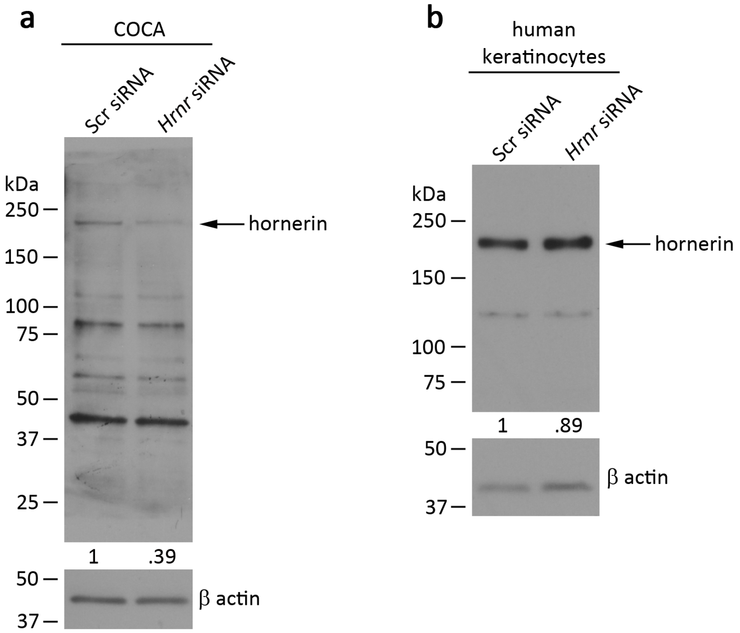
a
COCA
Scr siRNA
Hrnr siRNA
kDa
250
150
100
75
50
37
25
hornerin
1
.39
50
37
β actin
b
human
keratinocytes
Scr siRNA
Hrnr siRNA
kDa
250
150
100
75
hornerin
1
.89
50
37
β actin

Supplementary Figure 4. Mouse *Hrnr* siRNA does not significantly reduce human hornerin expression. (**a**) The murine epidermal keratinocyte cell line COCA was treated with either Scr siRNA or siRNA targeting murine *Hrnr* for 24hrs., lysed in RIPA buffer, and hornerin protein expression was determined by immunoblot. Beta actin expression was used as a loading control. Image depicts ~60% hornerin knockdown, as determined by densitometry, in *Hrnr* siRNA treated COCA cells compared to Scr siRNA control cells. (**b**) Hornerin was expressed at near equivalent levels (~90%) in human keratinocytes treated under similar siRNA conditions. Molecular weight is indicated in kilodlatons (kDa). Representative hornerin band indicated by arrow.

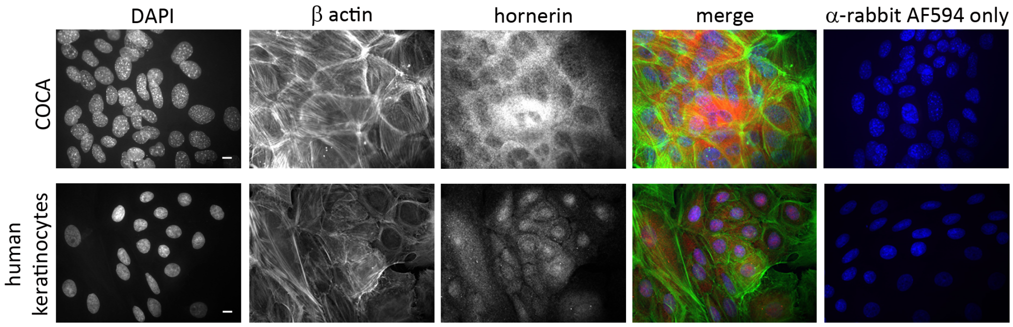
DAPI
β actin
hornerin
merge
α-rabbit AF594 only
COCA
human keratinocytes

Supplementary Figure 5. The anti-hornerin antibody utilized for indirect fluorescent microscopy of tumor sections cross-reacts with murine hornerin. COCA or human keratinocytes were seeded onto fibronectin coated coverslips and treated with 2mM $CaCl_2$ for two days, followed by incubation with anti-hornerin and anti-beta actin FITC primary antibodies. The samples were subsequently incubated with the secondary antibody anti-rabbit AF594 prior to mounting with Prolong Gold + DAPI. Representative images depict hornerin expression (red, merged image) in both COCA (top row) and human keratinocytes (bottom row). Scale bar = 10μm.

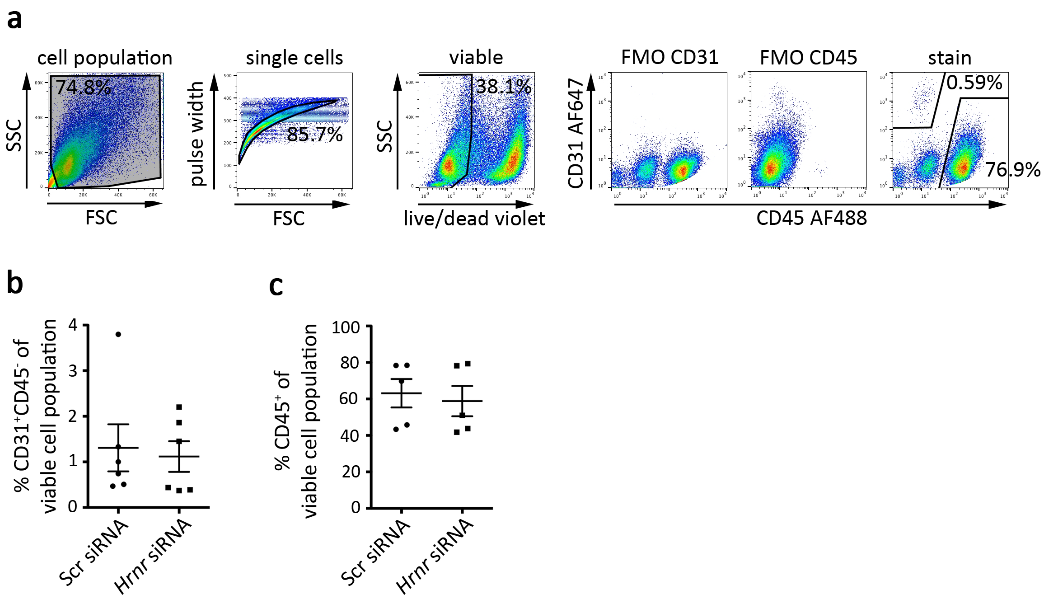
a
cell population
74.8%
SSC
FSC
single cells
85.7%
pulse width
FSC
viable
38.1%
SSC
live/dead violet
FMO CD31
FMO CD45
stain
0.59%
76.9%
CD31 AF647
CD45 AF488
b
% CD31+CD45- of viable cell population
Scr siRNA
Hrnr siRNA
c
% CD45+ of viable cell population
Scr siRNA
Hrnr siRNA

Supplementary Figure 6. Identification of tumor cell populations by fluorescence activated cell sorting (FACS). (**a**) Representative flow cytometry dot plots of single cell suspensions generated from sub-cutaneous L3.6pl xenografts. Displayed is the gating scheme utilized to identify $CD31^{+}CD45^{-}$ and $CD45^{+}$ cell populations. From left to right, the gated populations in each plot represent the tumor cell population, single cells in the tumor cell population, viable single cells, and the expression of CD31 and CD45 on viable single cells, respectively. Fluorescence minus one (FMO) plots for CD31 and CD45 are indicated. The gated populations in the stain panel are representative of the $CD31^{+}CD45^{-}$ and $CD45^{+}$ populations that were identified and sorted during FACS. (**b**) and (**c**) Graphs display the percentage of (**b**) $CD31^{+}CD45^{-}$ and (**c**) $CD45^{+}$ cells out of the total cell population that were analyzed and sorted during FACS. The number of tumor cell suspensions analyzed for each treatment = 6 ($CD31^{+}CD45^{-}$) and 5 ($CD45^{+}$). Graphs represent mean +/- SEM. Statistical comparisons by unpaired two-tailed t test.

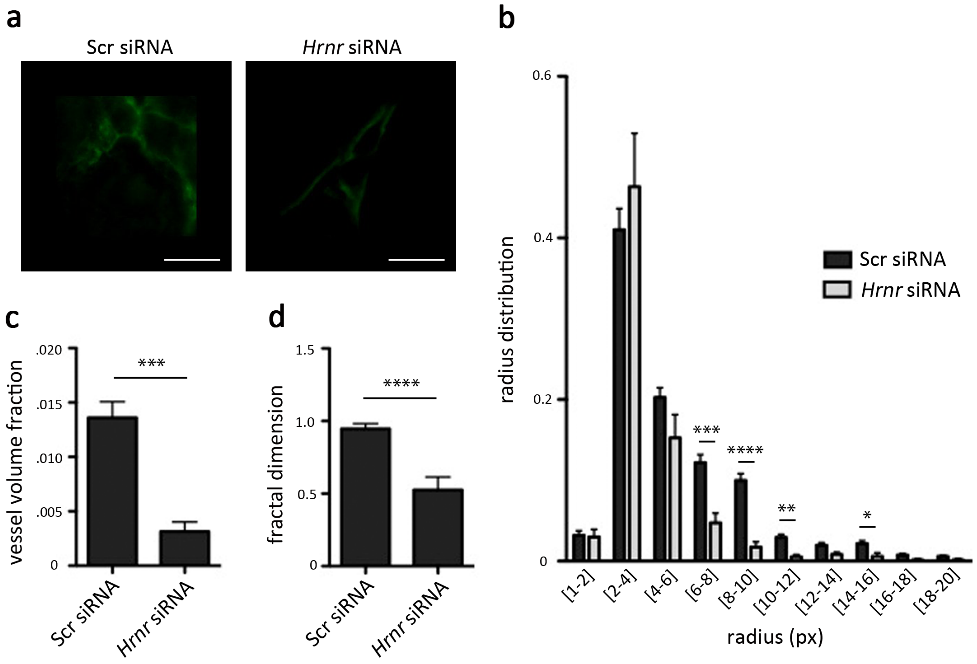
a
Scr siRNA
Hrnr siRNA
b
0.6
0.4
0.2
0
radius distribution
Scr siRNA
Hrnr siRNA
***
****
**
*
[1-2]
[2-4]
[4-6]
[6-8]
[8-10]
[10-12]
[12-14]
[14-16]
[16-18]
[18-20]
radius (px)
c
.020
.015
.010
.005
0
vessel volume fraction
***
Scr siRNA
Hrnr siRNA
d
1.5
1.0
0.5
0
fractal dimension
****
Scr siRNA
Hrnr siRNA

Supplementary Figure 7. The tumor vasculature of L3.6pl tumor bearing mice treated with a second unique set of pooled *Hrnr* siRNA (Origene) display a trend toward smaller radii, reduced volume fraction, and reduced fractal dimension compared to mice treated with Scr siRNA (Origene). (**a**) *Hrnr* siRNA or Scr siRNA were injected into L3.6pl-derived tumors on day 5 post-innoculation. A second round of siRNA treatment was completed three days later, and the tumors were harvested the following day (day 9 post-innoculation), fixed in formalin, embedded in paraffin, and sectioned (5μm) for immunofluorescence detection of CD34. Representative images of CD34 staining (green) in Scr siRNA (left) and *Hrnr* siRNA (right) treated tumors. Images displaying $CD34^+$ vasculature in Scr siRNA and *Hrnr* siRNA tumors were analyzed using RAVE software and the mean radii distribution in pixel units (**b**), vessel volume fraction (**c**), and fractal dimension (**d**) were calculated for each treatment group. Two tumors were analyzed/treatment group, N (images analyzed) = 103 (Scr siRNA), 27 (*Hrnr* siRNA). Scale bar = 10μm. Graphs represent mean +/- SEM. *P ≤ .05, **P ≤ .01, ***P ≤ .001, ****P ≤ .0001 by unpaired two-tailed t test.

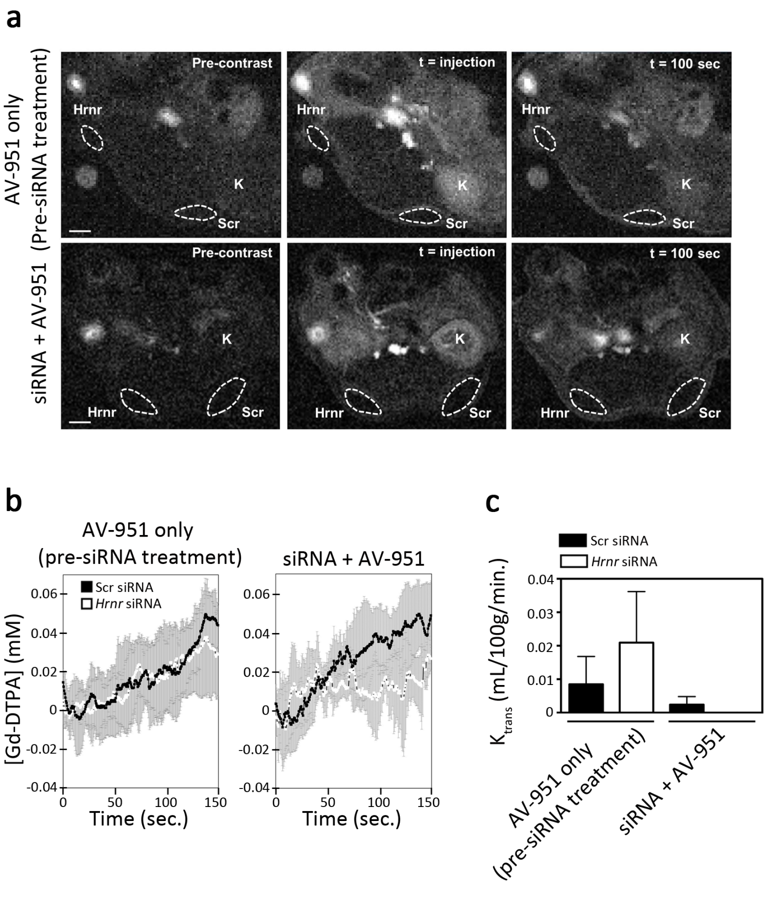
a
AV-951 only
(Pre-siRNA treatment)
siRNA + AV-951
Pre-contrast
t = injection
t = 100 sec
Hrnr
K
Scr
b
AV-951 only
(pre-siRNA treatment)
siRNA + AV-951
Scr siRNA
Hrnr siRNA
[Gd-DTPA] (mM)
Time (sec.)
c
Scr siRNA
Hrnr siRNA
K trans (mL/100g/min.)
AV-951 only
(pre-siRNA treatment)
siRNA + AV-951

Supplementary Figure 8. Dynamic contrast-enhanced magnetic resonance imaging of L3.6pl tumors treated with AV-951 or AV-951 + siRNA . (**a**) Representative axial time course images of gadolinium-DTPA (Gd-DTPA) uptake in AV-951 treated mice (top row) or mice treated with combination AV-951 and siRNA (bottom row). The kidney ("K") is labeled for reference. Scr siRNA and *Hrnr* siRNA tumors demarcated by a white dashed line. (**b**) The concentration of Gd-DTPA (mM) in tumors treated with AV-951 + Scr siRNA or AV-951 + *Hrnr* siRNA was plotted over time. (**c**) K_{trans} was calculated for AV-951 only (columns 1 and 2) and AV-951 + siRNA (columns 3 and 4). As before, the AV-951 only animals were imaged before treatment with siRNA (columns 1 and 2 pre-siRNA treamtnet; columns 3 and 4 AV-951 post siRNA treatment). N = 5 tumors/treatment group, except AV-951 only (column 2; N=4). Scale bar = 2mm. Graphs represent mean +/- SEM. Statistical comparisons by unpaired two-tailed t test.

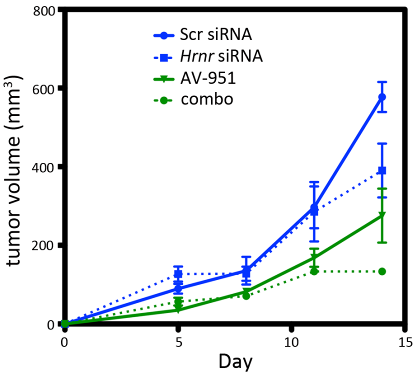

Scr siRNA
Hrnr siRNA
AV-951
combo
tumor volume (mm3)
800
600
400
200
0
0
5
10
15
Day

Supplementary Figure 9. L3.6pl tumor outgrowth. Complete outgrowth curves for the four treatment groups. N = 3 (Scr siRNA), N = 4 (*Hrnr* siRNA, AV-951, combo). Graphs represent mean +/- SEM.

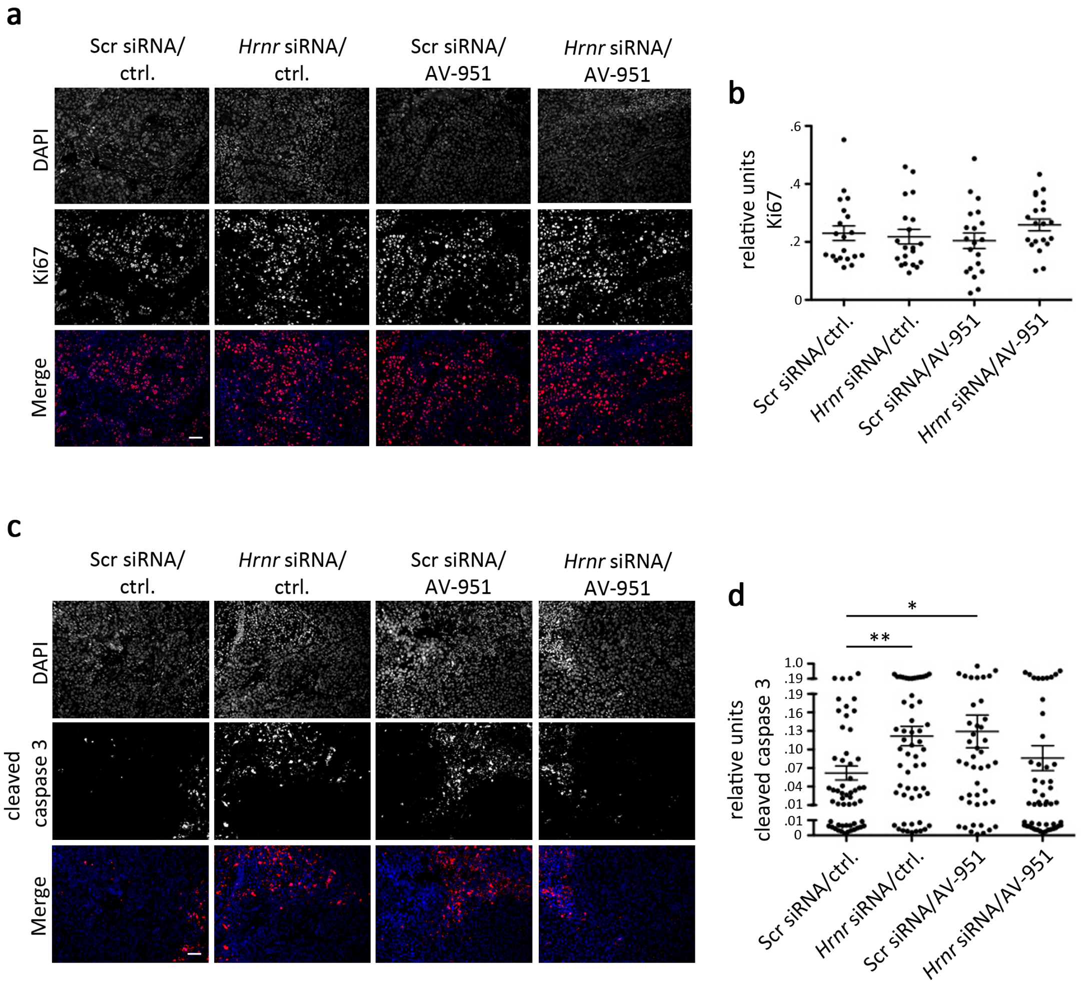

a
Scr siRNA/ ctrl.
Hrnr siRNA/ ctrl.
Scr siRNA/ AV-951
Hrnr siRNA/ AV-951
DAPI
Ki67
Merge
b
relative units Ki67
.6
.4
.2
0
Scr siRNA/ctrl.
Hrnr siRNA/ctrl.
Scr siRNA/AV-951
Hrnr siRNA/AV-951
c
Scr siRNA/ ctrl.
Hrnr siRNA/ ctrl.
Scr siRNA/ AV-951
Hrnr siRNA/ AV-951
DAPI
cleaved caspase 3
Merge
d
*
**
relative units cleaved caspase 3
1.0
.19
.19
.16
.13
.10
.07
.04
.01
.01
0
Scr siRNA/ctrl.
Hrnr siRNA/ctrl.
Scr siRNA/AV-951
Hrnr siRNA/AV-951

Supplementary Figure 10. Tumors from *Hrnr* siRNA or VEGFR inhibitor treated mice display similar expression of Ki67 and elevated cleaved caspase 3. Formalin fixed day 15 tumor sections from each of the four treatment groups were deparraffinized, rehydrated, and incubated with an anti-Ki67 or anti-cleaved caspase 3 primary antibody. Following a secondary antibody incubation with anti-rabbit AF594, the sections were mounted in Prolong Gold + DAPI and images were acquired at 20x magnification. (**a**) Representative images from Day 15 tumor sections for DAPI (top), Ki67 (middle), and merged image (bottom; Ki67 (red), DAPI (blue)). (**b**) Data presented for Ki67 expression are from the analysis of 10 images/tumor from two tumors/treatment group. (**c**) Representative images from day 15 tumor sections for DAPI (top), cleaved caspase 3 (middle), and merged image (bottom; cleaved caspase 3 (red), DAPI (blue)). (**d**) A minimum number of 10 images/tumor section from four tumor sections/treatment group were analyzed for cleaved caspase 3 expression. Total number of images analyzed/group, N= 55 (Scr siRNA/ctrl.), 55 (*Hrnr* siRNA/ctrl.), 44 (Scr siRNA/AV-951), 51 (*Hrnr* siRNA/AV-951). Scale bar = 50µm. Graphs represent mean +/- SEM. *P ≤ .05, **P ≤ .01 by umpaired two tailed t test.

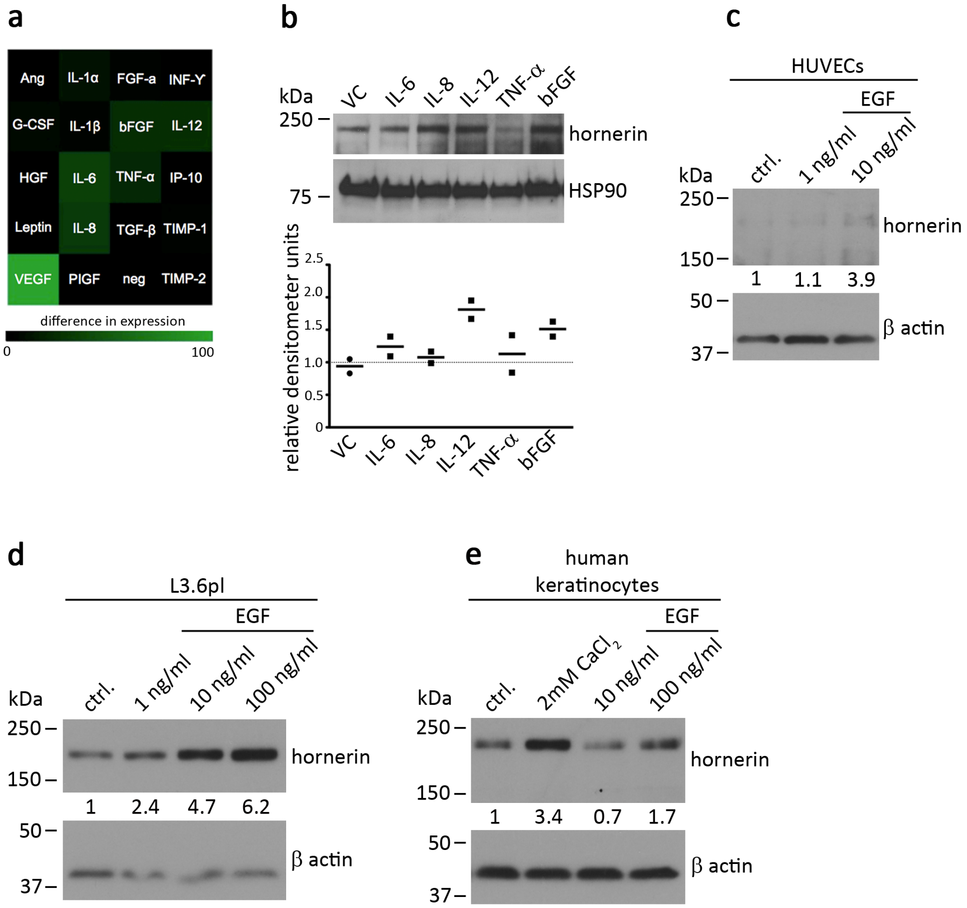
a
Ang
IL-1α
FGF-a
INF-Y
G-CSF
IL-1β
bFGF
IL-12
HGF
IL-6
TNF-α
IP-10
Leptin
IL-8
TGF-β
TIMP-1
VEGF
PIGF
neg
TIMP-2
difference in expression
0
100
b
kDa
VC
IL-6
IL-8
IL-12
TNF-α
bFGF
250
hornerin
75
HSP90
relative densitometer units
2.5
2.0
1.5
1.0
0.5
0
VC
IL-6
IL-8
IL-12
TNF-α
bFGF
c
HUVECs
EGF
kDa
ctrl.
1 ng/ml
10 ng/ml
250
hornerin
150
1
1.1
3.9
50
β actin
37
d
L3.6pl
EGF
kDa
ctrl.
1 ng/ml
10 ng/ml
100 ng/ml
250
hornerin
150
1
2.4
4.7
6.2
50
β actin
37
e
human keratinocytes
EGF
kDa
ctrl.
2mM CaCl2
10 ng/ml
100 ng/ml
250
hornerin
150
1
3.4
0.7
1.7
50
β actin
37

Supplementary Figure 11. The regulation of hornerin expression by factors present in the tumor secretome. (**a**) Heatmap of the difference in expression of TCM compared to VC media as measured by a Human Angiogenesis Antibody Array. "neg" refers to a negative control built into the assay by the manufacturer. (**b**) Representative western blot of the lysates of HUVECs treated with the top 5 differentially expressed growth factors. HSP90 is presented as a loading control. The graph displays the relative densitometry of 2 separate experiments measured as fold HRNR expression over HSP90. Bar represent the mean value. (**c**) HUVECs were plated at a density of $0.15x10^6$ cells/well in 6 well cluster plates in complete media, starved of EGF for 14hrs., and subsequently treated with EGF at the indicated concentrations. Hornerin expression was determined by immunoblot after 24hrs. treatment. Beta actin was utilized as a loading control. (**d**) L3.6pl cells were plated in complete media in 6 well cluster plates at a density of $0.2x10^6$ cells/well. On day 2, the cells were replenished with serum free media and serum starved overnight, upon which vehicle control or EGF was added at the indicated concentrations. The cells were subsequently lysed in RIPA buffer at 48hrs. and lysate preparations were subjected to immunoblot detection of hornerin. Beta actin is presented as a control. (**e**) Human keratinocytes were plated at a density of $0.1x10^6$ cells/well in 6 well plates and treated on day 2 with either EGF or 2mM $CaCl_2$. Lysate preparations were generated 48hrs. post-treatment for immunoblot detection of hornerin. Beta actin is presented as a control. Molecular weight is indicated in kilodlatons (kDa).

		Frequency (Number)
Age (years)	50 - 60<br>60 - 70<br>70 - 80<br>> 80	50% (5)<br>30% (3)<br>10% (1)<br>10% (1)
Gender	Male<br>Female	40% (4)<br>60% (6)
Race	White	100% (10)
Tumor Grade	1 - Well Differentiated<br>2 - Moderately Differentiated<br>3 - Poorly Differentiated	10% (1)<br>20% (2)<br>70% (7)

Supplementary Table 1. De-identified PDAC patient and tumor characteristics used in assessment of hornerin expression in tumor associated endothelium.
